# Survival Following Colorectal Cancer Surgery in Low‐ and Middle‐Income Countries: A Systematic Review and Meta‐Analysis

**DOI:** 10.1002/jso.70235

**Published:** 2026-03-15

**Authors:** Oluwasegun Afolaranmi, Thomas M. Diehl, Sehar Salim Virani, Qiuyu Yang, Leslie Christensen, Kenechukwu Okwunze, Adel H. Khan, Nabiha Akhlaq Mughal, Daniel Schroeder, Sheida Pourdashti, Sadaf Khan, Syed Nabeel Zafar

**Affiliations:** ^1^ Department of Surgery Mayo Clinic Rochester Minnesota USA; ^2^ Department of Surgery University of Wisconsin–Madison Madison Wisconsin USA; ^3^ Department of Surgery Aga Khan University Karachi Sindh Pakistan; ^4^ Ebling Library for the Health Sciences, School of Medicine and Public Health University of Wisconsin Madison Wisconsin USA; ^5^ Gillings School of Global Public Health University of North Carolina Chapel Hill North Carolina USA; ^6^ Department of Anesthesiology and Critical Care Houston Methodist Hospital Houston Texas USA; ^7^ The University of Texas Health Science Center San Antonio Texas USA; ^8^ Department of Radiology Aurora St. Luke's Medical Center Milwaukee Wisconsin USA; ^9^ School of Medicine and Public Health University of Wisconsin Madison Wisconsin USA; ^10^ University of Wisconsin Carbone Cancer Center Madison Wisconsin USA

**Keywords:** colorectal cancer, low‐ and middle‐income countries, survival

## Abstract

**Background and Objectives:**

Surgery is the mainstay of treatment for localized colorectal cancer (CRC); however, little is known about survival outcomes following CRC surgery in low‐ and middle‐income countries (LMICs). Here, we examine the available data on long‐term outcomes following CRC resections in LMICs.

**Methods:**

A systematic review and meta‐analysis were conducted on primary research studies reporting survival data after CRC resection with curative intent in LMICs. Disease‐free survival (DFS) and overall survival (OS) data were extracted, and random effects modelling was used to estimate the pooled survival rates.

**Results:**

One hundred and fifty‐four studies representing 20,589 CRC patients were analyzed. Notably, only 27 (20%) of the 137 LMICs were represented in the literature. The pooled 5‐year OS estimate was 88% (95% CI: 77–95), 76% (69–81), and 57% (49–64) for Stages I, II, and III, respectively. 5‐year DFS estimates were 82% (71–90), 76% (67–84), and 59% (51–65) for stages I, II, and III, respectively. Combined OS and DFS estimates for all three stages were 76% and 69%, respectively. Survival rates varied considerably across the included studies and between income groups.

**Conclusions:**

With the rising incidence of CRC globally, our work highlights the dearth of data on long‐term outcomes following CRC operations in most LMICs and emphasizes the urgent need for research capacity building.

## 1 Introduction

Colorectal cancer (CRC) is the second most common cause of cancer‐related mortality globally, accounting for nearly a million deaths every year [1]. With increasing incidence, CRC presents a significant global health challenge, particularly in low‐ and middle‐income countries (LMICs). While the incidence of colorectal cancer is up to four times higher in high‐income countries, the case fatality rate is higher in LMICs [1, 2].

Surgery is the mainstay of treatment for localized CRC; however, survival after surgery is impacted by various individual and systemic factors. Disparities in healthcare systems, as well as sociodemographic factors, hugely influence surgical outcomes and survival rates. In LMICs, challenges such as insufficient understanding of disease biology, limited access to advanced surgical techniques and infrastructure, and inadequate postoperative care negatively impact postoperative outcomes and survival [3, 4]. This is further complicated by the paucity of robust and systematic surgical outcomes data collection and tracking systems, impairing quality improvement efforts [5].

To develop effective policies to address the high burden of CRC, regular tracking and reporting of outcomes data are important. Although several small to medium‐scale studies have been conducted on outcomes following CRC surgery in different LMICs, there is currently no comprehensive analysis of survival data on this subject across LMICs. Here, we provide a systematic review and meta‐analysis of the current literature on survival following CRC resection with curative intent in LMICs, synthesizing the available data and highlighting critical evidence gaps.

## 2 Methods

We conducted a systematic review and meta‐analysis of original research articles with data on survival outcomes following CRC resection in LMICs. Our review followed the Preferred Reporting Items for Systematic Reviews and Meta‐Analyses (PRISMA) reporting guidelines [6], and the study protocol was registered with the International Prospective Register of Systematic Reviews (PROSPERO 2021 CRD42021262906).

### 2.1 Search Strategy

The review team collaborated with a research librarian (LC) to develop a comprehensive search for primary research studies related to survival outcomes following colon and rectal cancer surgery in low‐ and middle‐income countries (LMICs). The terms for LMICs were adapted from a filter developed by the Cochrane Effective Practice and Organization of Care Group based on the 2019 World Bank list of low‐ and middle‐income countries [7]. Searches were conducted on June 1, 2021, and again on May 24, 2022, in the following databases: PubMed, Scopus (Elsevier), Global Index Medicus, and Web of Science (Clarivate) as a multi‐file search of Science Citation Index‐Expanded and Emerging Sources Citation Index. A date limit of January 1, 2005, to the time of search was applied to capture morbidity and mortality results from more modern, relevant surgical procedures. The following were excluded from results prior to screening using search hedges: Animal studies and non‐primary research studies with fewer than 50 participants. In Scopus, results were limited to Embase records. No language filter was applied to the results. Search results were downloaded to the citation management software, EndNote, and underwent manual deduplication by a librarian. Unique records were uploaded to a screening platform (Covidence) for independent review by team members using pre‐determined inclusion/exclusion criteria. The complete search strategy is available in the Supplemental Material.

### 2.2 Inclusion and Exclusion Criteria

All primary studies on adults (> 18 years) undergoing surgical resection of colon and rectal cancers with curative intent in LMICs were included. Low‐ and middle‐income countries were defined according to the 2020 World Bank income group categories [8]. Only primary research articles with survival outcomes (Disease Free Survival [DFS] and Overall Survival [OS]) were selected. We specifically excluded articles involving pediatric populations (< 18 years), non‐primary colorectal cancers, benign disease, stage IV colorectal cancer, and endoscopic/transanal resection only. In addition, letters to the editor and editorials, review manuscripts without primary research, case reports/series with fewer than 20 patients, studies of palliative surgical therapy, and studies without full text availability in English were excluded. Studies from China dominated the published literature and were excluded to avoid skewing the results.

### 2.3 Study Screening and Selection

Study screening was completed using Covidence systematic review software (Veritas Health Innovation, Melbourne, Australia; available at www.covidence.org) [9]. Once all articles were loaded into the Covidence database, review and study selection were carried out in two distinct phases: first, title and abstract screening, followed by full‐text review. In both phases, each article was assessed for inclusion by two independent reviewers (Thomas M. Diehl, Sehar Salim Virani, Adel H. Khan, Nabiha Akhlaq Mughal), and a third reviewer (Oluwasegun Afolaranmi) resolved conflicts. Articles with two votes for inclusion advanced to the next phase.

### 2.4 Quality Assessment

The included articles were reviewed for quality using the appropriate NIH Study Quality Assessment tool [10]. Study quality was assessed by two independent reviewers. Individual items on the quality tool received a score of one (1) and were summed to make the final “quality score” for each study. Each study was assigned a grade of ‘poor’, ‘fair’, or ‘good’ based on scoring within the first, second, or third tertile, respectively. Studies that received a ‘fair’ or ‘good’ grade were included in the meta‐analysis.

### 2.5 Data Extraction and Analysis

For all articles meeting inclusion criteria, we extracted data on study design, demographics (e.g., country of origin, mean population age), TNM stage, and survival. Dukes' staging system was less frequently reported and was converted to TNM stage via National Institute for Clinical Excellence (NICE) guidelines. Specifically, DFS and OS were extracted from full‐text and Kaplan‐Meier curves in 6‐month intervals to a maximum of 60 months. Each manuscript reaching the data extraction phase was reviewed initially by a single team member, with selected data points entered into the Covidence database. Data extraction was completed by two independent team members, then reviewed by a third team member before analysis. Survival data were manually extracted from Kaplan‐Meier curves using “Plotdigitizer” online software (Available at https://plotdigitizer.com).

### 2.6 Statistical Analysis

All collected data were exported from Covidence for analysis in R statistical software (R 4.4.2). Descriptive analyses were conducted for several study characteristics such as study population, study type, disease stage, and quality rating. Extracted data were categorized by World Bank country income designation (low‐income, lower‐middle‐income, upper‐middle‐income) and weighted to report pooled DFS and OS at 6‐month intervals. The random‐effects model with Logit transformation was used to estimate the pooled survival proportions, accounting for between‐study heterogeneity. Subgroup analysis was done to compare DFS and OS across different income levels. Data are presented as proportions and 95% confidence intervals. *p*‐values < 0.05 were considered statistically significant.

## 3 Results

**Figure 1:**
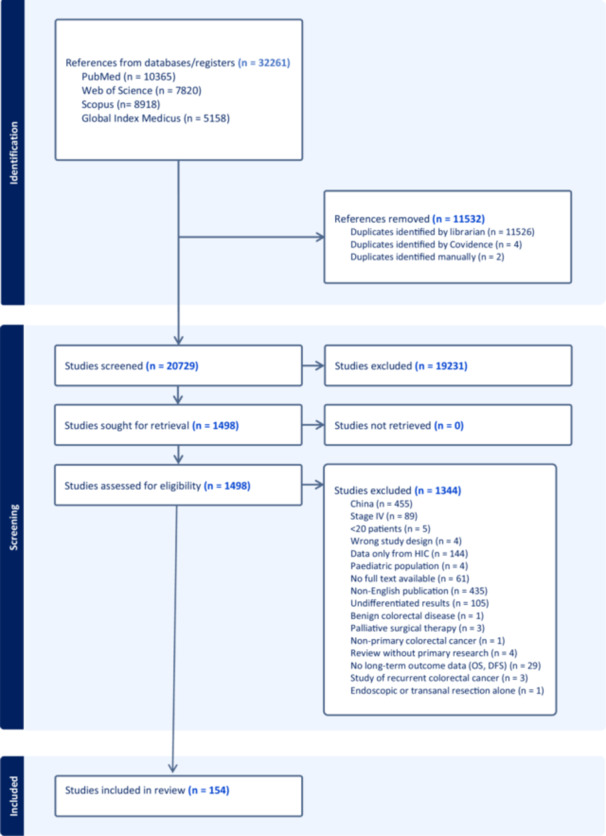
PRISMA flow diagram.

**Figure 2:**
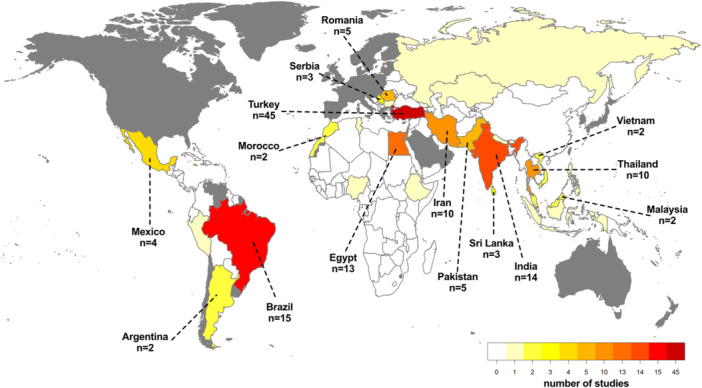
Heatmap showing the distribution of identified studies from LMICs included in the meta‐analysis. LMICs with at least 2 studies are annotated on the map. High‐income countries (HICs) are colored grey and not included.

Of the 20,731 articles that were assessed for eligibility, 154 studies met the inclusion criteria, representing 20,589 colorectal cancer patients across 27 LMICs. The PRISMA flow diagram is presented in Figure 1. 98 (63.6%) of the included studies were from upper‐middle‐income countries (upperMIC), 55 (35.7%) from lower‐middle‐income countries (lowerMIC), and only one was from a low‐income country (LIC) ‐ Ethiopia. Notably, only 27 (20%) of the 137 LMICs were represented in the literature (Figure 2 and Table 3.1). Turkey accounted for the highest frequency of included articles, with 45 (29%) studies (Figure 2). Most (60%) of the studies were retrospective cohort designs, and only four (4) were randomized controlled trials. Study and population characteristics by World Bank income level are presented in Table 1.

**Table 1:** Study and population characteristics and representation by World Bank income level.

Study characteristic	Low income	Lower middle income	Upper middle income	Overall
No of studies, *n* (%)	1 (0.6)	55 (35.7)	98 (63.6)	154
Study Type, *n* (%)				
Randomized controlled		2 (50.0)	2 (50.0)	4
Non‐randomized trial		3 (37.5)	5 (62.5)	8
Prospective cohort study		10 (43.5)	13 (56.5)	23
Retrospective cohort study	1 (1.1)	31 (33.3)	61 (65.6)	93
Case‐control study		1 (25.0)	3 (75.0)	4
Cross‐sectional study		5 (62.5)	3 (37.5)	8
Case series		3 (23.1)	10 (76.9)	13
Other			1 (100)	1
NIH Quality Score, *n* (%)				
Good	1 (0.8)	42 (32.6)	86 (66.7)	129
Fair		13 (54.2)	11 (45.8)	24
Poor				

Population Characteristic	Low Income	Lower Middle Income	Upper Middle Income	Missing*
TNM Stage, mean (SD)				
I	5 (N.A)	37.8 (48.6)	35.5 (38.6)	64.2
II	57 (N.A)	81.1 (73.7)	90.5 (79.1)	59.5
III	22 (N.A)	107.5 (100.0)	104.1 (120.4)	57.4
Mean age (SD)				
	45.9 (NA)	53.5 (7.0)	59.9(4.7)	27.0

* Represents the percentage of missing values for the respective variable.

Using the NIH Study Quality Assessment tool, 16% of the included studies were rated “fair”, and the majority (84%) were rated “Good” (Table 1). Notably, there was no statistically significant difference in ratings between studies from upper‐middle or lower‐middle‐income countries (*p* = 0.062, Fisher's Exact Test).

### 3.1 Disease Free Survival (DFS)

**Figure 3:**
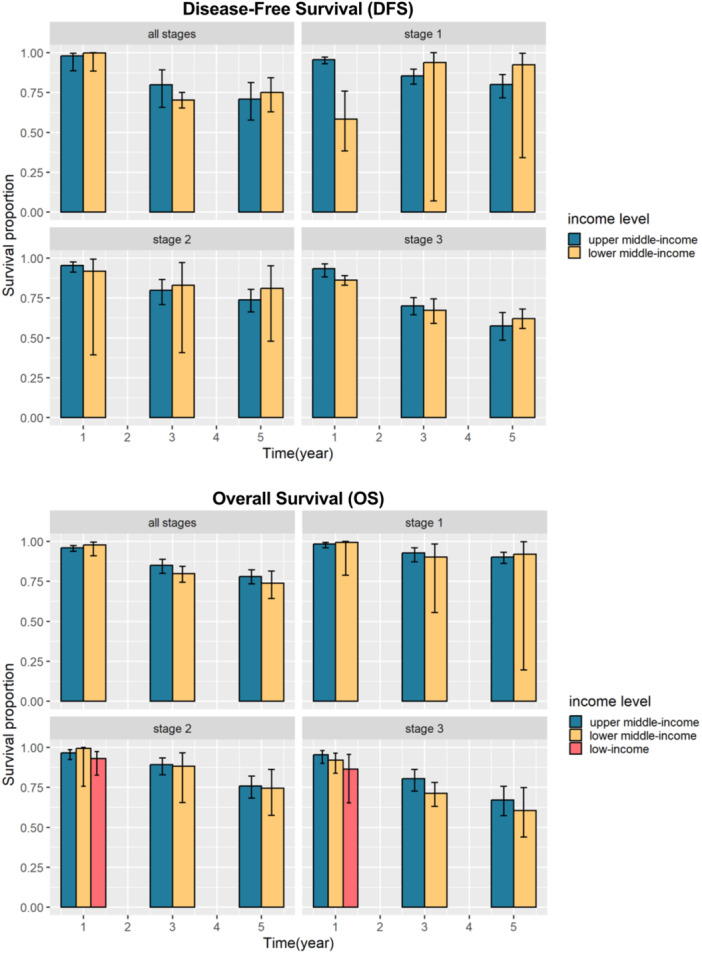
Pooled Disease‐Free Survival (DFS) and Overall Survival (OS) estimates by cancer stage and income category.

Using a random effects model, the pooled DFS estimate following cancer surgery for stage I–III colorectal cancer in LMICs was 98% (95% CI: 95–99) at 1 year, 77% (71–82) at 3 years, and 70% (62–76) at 5 years. 5‐year DFS estimates were 82% (71–90), 76% (67–84), and 59% (51–66) for stage I, II, and III disease, respectively. Across all three cancer stages included, 5‐year DFS was 73% (60–82) in lower‐middle‐income countries and 68% (59–76) in upper‐middle‐income countries (*p* = 0.5508). DFS estimates by income group and cancer stage are summarized in Figure 3 and Table 3.1.

### 3.2 Overall Survival (OS)

**Figure 4:**
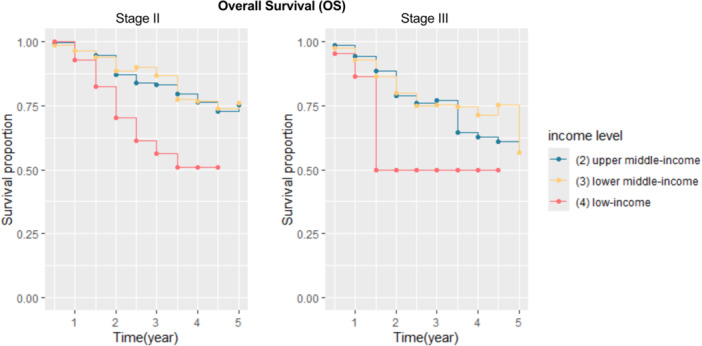
Kaplan‐Meier curves showing differences in pooled OS rates between middle‐ and low‐income countries for stage II and III CRC cancer.

The pooled OS estimates for stage I–III colorectal cancer were 97% (94–98) at 1 year, 82% (77–86) at 3 years, and 76% (72–79) at 5 years. 5‐year OS estimate was 88% (77–95) for Stage I, 76% (69–81) for Stage II, and 57% (49–64) for Stage III disease. The 5‐year OS was 72% (64–79) in lower‐middle‐income countries and 77% (72‐81) in upper‐middle‐income countries (*p* = 0.2097). Detailed OS data by income group and cancer stage at 1‐, 3‐, and 5‐year time points are presented in Figure 3 and Table 3.1. Where data is available, we compared OS rates between studies from middle‐ and low‐income countries at 6‐month intervals. Across multiple time points, survival rates were significantly lower in the low‐income group compared to lower‐middle‐ and upper‐middle‐income countries (Figure 4).

## 4 Discussion

Recent estimates show that the burden of CRC is expected to increase in the next decades [11, 12], with LMICs projected to be most impacted. This underscores the need for high‐quality data to guide in setting priorities and monitoring cancer control efforts. Our review highlights the limited data on survival following surgery for CRC in LMICs, with 3 countries (Turkey, Brazil, and India) accounting for half of all available studies, and no published data found for 105 (79.5%) countries during the review period. Notably, there is a paucity of data from LICs, with Ethiopia being the only country in this category represented in our study. Most of the studies are retrospective cohort studies, with randomized controlled trials contributing the smallest fraction. Findings from our pooled survival outcomes at 1 year stratified by cancer stage reveal similarities with data published from HICs [13, 14]. However, marked differences exist when comparing outcomes at later timepoints.

Routine systematic collection and audit of data on survival rates are instrumental in evaluating the quality of cancer care and implementing strategies to improve outcomes [15]. However, in many LMICs, this remains a significant challenge due to systemic barriers such as inadequate funding [16, 17, 18], limited research infrastructure, workforce shortages, and a strained health system that need to be addressed in order to improve data collection and strengthen cancer surveillance. To build research capacity in LMICs, there must be improved focus on (1) strengthening research infrastructure by providing research equipment such as internet enabled devices, data collection tools, statistical software, and access to peer‐reviewed journals; (2) investing in mentorship and training by organizing research clubs, workshops and courses on research methods, ethics, biostatistics, and grant writing, or supporting postgraduate degree programs, all of which can be done virtually or physically; (3) building institutional and regional collaborations that encourage local‐agenda setting to address pressing health needs; (4) creating sustainable funding mechanism by pooling funds from international bodies, government budgetary allocations, and the private sector [19, 20].

Initiatives, such as the H3 Africa [21], which supports genomic research in Africa, the African Cancer Registry Network (AFCRN) [22], which coordinates cancer surveillance in sub‐Saharan Africa, and the National Cancer Grid of India [23], which facilitates exchange programs and collaborative research across cancer centers in India, represent successful programs at regional, subregional, and national levels that have sustainably built research capacity. The Lancet Oncology commission on cancer in sub‐Saharan Africa [24] highlighted the critical role of high‐quality data in planning cancer control programs and improving outcomes over time. The commission further provided actionable recommendations on enhancing data reporting, many of which can be expanded for use by other LMICs. Implementing these recommendations is important, as without robust data systems and the data‐driven policies they enable, policymakers lack the evidence base needed to design targeted interventions, allocate resources efficiently, and monitor progress.

Our analysis found that the pooled 5‐year DFS rates in LMICs were consistently lower than reports from HICs. For example, a study by Lee and colleagues [25] involving 204 patients who underwent surgery for CRC in Korea reported higher 5‐year DFS rates for both patients with and without postoperative complications (77.4% and 85.6%, respectively) compared to our pooled analysis (73% in low‐middle and 68% in upper‐middle income countries). Similarly, a large multicenter Dutch study of patients undergoing surgery for CRC [26] reported superior 5‐year DFS rates across all stages, including a 9 percentage points difference for patients with stage I disease (91.1% vs. 82% in our analysis). These differences likely reflect disparities in access to multidisciplinary cancer care, adjuvant therapies, and postoperative follow‐up between LMICs and HICs.

Compared to upper‐middle‐income countries, our analysis found no difference in the OS rates observed in low‐middle‐income countries, and this was consistent across all cancer stages and at all timepoints. Notably, we identified studies from several LMICs that reported similar survival rates to HICs across various stages. In a study conducted in Chile [27], which stratified OS rates by type of health insurance as a proxy for socioeconomic strata, patients from the highest socioeconomic group achieved 5‐year survival rates similar to those reported in Sweden and Finland [28]. In contrast, a wide disparity in outcome was observed between patients in the highest and lowest socioeconomic classes, with pooled 5‐year OS of 64% and 31%, respectively. While socioeconomic inequalities in survival following surgery for CRC in HICs [29, 30, 31] have been documented, the magnitude of disparity appears greater in LMICs. Similar socioeconomic disparities in survival have been described in other cancers, including breast and cervical cancer [32, 33], underscoring their profound impact on cancer outcomes in LMICs.

Worse stage‐for‐stage CRC outcomes in LMICs arise from an interplay of systemic factors that revolve around access to care and delivery of care. These factors contribute to varying degrees across different health systems in LMICs. Barriers linked to ‘access to care’ include high out‐of‐pocket costs of cancer care, limited insurance coverage, geographical isolation faced by patients residing far from cancer centers [34], reduced access to surgical care [35], and a shortage of essential chemotherapeutic agents for CRC. On the other hand, factors linked to the ‘delivery of care’ include a low prevalence of guideline‐concordant care [36], the circulation of substandard chemotherapy [37], and possibly, a low uptake of post‐treatment colonoscopy surveillance to detect and manage recurrences promptly. Some reports suggest a more aggressive CRC presentation among populations in LMICs [38, 39], and this may also contribute to the worse outcomes in such settings. However, further studies are needed to enhance our understanding of this relationship.

Although national and regional estimates have been reported [40, 41], this review is the first meta‐analysis of colorectal cancer survival rates that pooled data across LMICs on various continents, to the best of our knowledge. This provides a broader overview and enhances the generalizability of our findings. Our study has several limitations, including the possibility of publication bias arising from ‘selective‐reporting’ whereby institutions and countries with better‐resourced healthcare systems and stronger research infrastructure are more likely to generate and publish their survival data, while under‐resourced settings, which may have worse outcomes, are less represented in the literature. This may ultimately lead to an over‐estimation of survival rates in LMICs. Also, colon cancers and rectal cancers are distinct entities that differ in treatment approaches, combining survival estimates for these two cancers and interpreting them as one may obscure important site‐specific differences. Furthermore, the heterogeneity of included studies from LMICs with diverse healthcare systems and surgical practices, variations in staging systems and treatment protocols, differences in analytical approach (such as whether emergent cases presenting with perforation and obstruction were included and how cases with postoperative complications resulting in delayed adjuvant therapy were handled), and the quality/completeness of data from some LMICs may introduce further bias to our findings.

## 5 Conclusions

Our work highlights the dearth of data on long‐term outcomes following colorectal cancer surgery in most LMICs. From the available data, we identify notable disparities in survival rates between LMICs and published reports on HICs. Strengthening the routine collection and reporting of robust cancer survival data in LMICs and leveraging these data to formulate evidence‐based policies will be pivotal in setting priorities and improving cancer outcomes.

## Conflicts of Interest

The authors declare no conflicts of interest.

## 6 Synopsis

This study systematically reviews long‐term survival outcomes after colorectal cancer (CRC) surgery in low‐ and middle‐income countries (LMICs), presenting pooled survival data from 154 studies with over 20,000 patients. Our findings reveal wide variability and limited country representation, underscoring a critical need for improved research infrastructure and data collection in LMICs.

## Supporting information

Supplemental_File.

## Data Availability

Data sharing not applicable to this article as new datasets were generated. The comprehensive search strategy for the systematic review is provided in the Supplemental file.
