# Supplementary material for: Survival Following Colorectal Cancer Surgery in Low‐ and Middle‐Income Countries: A Systematic Review and Meta‐Analysis
Source: J Surg Oncol. 2026 Mar 15;133(7):872–80. doi: 10.1002/jso.70235 (PMC13352226; doi:10.1002/jso.70235)
Supplement: Supplementary file 1 — Supplemental_File. [file JSO-133-872-s001.docx]

**Supplemental File**

**Supplemental Table 1.**

| **Country** | **HDI income group** | **Number of studies (n)** |
| --- | --- | --- |
| Turkey | UpperMIC | 45 |
| Brazil | UpperMIC | 17 |
| India | LowerMIC | 14 |
| Egypt | LowerMIC | 13 |
| Iran | LowerMIC | 10 |
| Thailand | UpperMIC | 10 |
| Pakistan | LowerMIC | 6 |
| Mexico | UpperMIC | 6 |
| Romania | UpperMIC | 5 |
| Serbia | UpperMIC | 4 |
| Sri Lanka | LowerMIC | 3 |
| Morocco | LowerMIC | 2 |
| Vietnam | LowerMIC | 2 |
| Argentina | UpperMIC | 2 |
| Jamaica | UpperMIC | 2 |
| Malaysia | UpperMIC | 2 |
| Ethiopia | LIC | 1 |
| Indonesia | LowerMIC | 1 |
| Nepal | LowerMIC | 1 |
| Nigeria | LowerMIC | 1 |
| Philippines | LowerMIC | 1 |
| Tunisia | LowerMIC | 1 |
| Bulgaria | UpperMIC | 1 |
| Kazakhstan | UpperMIC | 1 |
| Lebanon | UpperMIC | 1 |
| Peru | UpperMIC | 1 |
| Russia | UpperMIC | 1 |

HDI- Human Development Index; UM- Upper middle-income; LM- Lower middle-income; L- Low-income.

**Supplemental Table 2.**

| Disease-free survival by World Bank Income Level and Cancer Stage | | | | | | |
| --- | --- | --- | --- | --- | --- | --- |
|  | **Low Income** | | **Lower Middle Income** | | **Upper Middle Income** | |
|  | percentage (95% CI) | N | percentage (95% CI) | N | percentage (95% CI) | N |
| 1-year DFS, percentage (95% CI) | **NA** |  | **99 (90-100)** |  | **97 (93-99)** |  |
| I |  |  | 58 (38-76) | 24 | 96 (95-98) | 592 |
| II |  |  | 92 (39-99) | 177 | 93 (89-95) | 1266 |
| III |  |  | 96 (94-98) | 454 | 92 (88-95) | 1165 |
| 3-year DFS, percentage (95% CI) | **NA** |  | **72 (67-77)** |  | **80 (71-87)** |  |
| I |  |  | 89 (44-99) | 84 | 86 (78-91) | 600 |
| II |  |  | 85 (57-96) | 472 | 80 (74-85) | 1323 |
| III |  |  | 79 (70-86) | 767 | 68 (63-73) | 1886 |
| 5-year DFS, percentage (95% CI) | **NA** |  | **73 (60-82)** |  | **68 (59-76)** |  |
| I |  |  | 92 (34-100) | 87 | 81 (72-87) | 537 |
| II |  |  | 83 (80-86) | 575 | 78 (75-80) | 1190 |
| III |  |  | 62 (56-68) | 246 | 62 (59-76) | 1140 |

| Overall survival by World Bank Income Level and Cancer Stage | | | | | | |
| --- | --- | --- | --- | --- | --- | --- |
|  | **Low Income** | | **Lower Middle Income** | | **Upper Middle Income** | |
|  | percentage (95% CI) | N | percentage (95% CI) | N | percentage (95% CI) | N |
| 1-year OS, percentage (95% CI) |  |  | **97 (91-99)** |  | **97 (94-98)** |  |
| I |  |  | 99 (90-100) | 485 | 98 (96-99) | 662 |
| II | 93 (83-97) | 57 | 97 (88-99) | 853 | 96 (93-98) | 1544 |
| III | 86 (65-96) | 22 | 93 (86-97) | 1458 | 94 (89-97) | 1425 |
| 3-year OS, percentage  (95% CI) |  |  | **81 (75-86)** |  | **82 (75-87)** |  |
| I |  |  | 93 (72-98) | 521 | 93 (90-96) | 670 |
| II | 56 (43-68) | 57 | 87 (74-94) | 1071 | 83 (76-88) | 1578 |
| III | 50 (30-70) | 22 | 75 (73-77) | 1621 | 77 (75-79) | 2115 |
| 5-year OS, percentage  (95% CI) |  |  | **72 (64-79)** |  | **77 (73-81)** |  |
| I |  |  | 92 (49-99) | 496 | 88 (76-95) | 968 |
| II |  |  | 76 (60-87) | 1215 | 75 (67-82) | 2200 |
| III |  |  | 57 (46-67) | 787 | 57 (46-66) | 2023 |

**Supplemental Table**

**Supplement: complete search strategy**

**Original Searches 6/1/21**

**PubMed (NLM)
9209 Results**((“Intestine, Large”[mesh:noexp] OR “Colon”[mesh] OR “Cecum”[mesh:noexp] OR “Rectum”[mesh] OR (large-intestine*[tiab] OR large-bowel*[tiab] OR cecum*[tiab] OR caecum*[tiab] OR coecum*[tiab] OR colorect*[tiab] OR colon*[tiab] OR rectum*[tiab] OR rectal[tiab] OR sigmoid*[tiab] OR abdominoperineal*[tiab] OR ((flexur*[tiab]) AND (colon*[tiab] OR spleen*[tiab] OR splenic[tiab] OR hepatic[tiab])) OR ((abdomen*[tiab] OR abdomin*[tiab]) AND (perineal*[tiab])))) AND ("Neoplasms"[mesh] OR (neoplas*[tiab] OR cancer*[tiab] OR adenocancer*[tiab] OR adenocarcin*[tiab] OR adenoma*[tiab] OR carcino*[tiab] OR malignan*[tiab] OR tumor*[tiab] OR tumour*[tiab] OR metasta*[tiab] OR cyst*[tiab] OR growth*[tiab] OR lesion*[tiab]))) AND ("Colectomy"[Mesh] OR “Proctectomy”[mesh] OR (surger*[tiab] OR surgery[sh] OR surgical*[tiab] OR resection*[tiab] OR debulk*[tiab] OR excision*[tiab] OR colectom*[tiab] OR hemicolectom*[tiab] OR proctectom*[tiab] OR proctocolectom*[tiab] OR coloproctectom*[tiab])) AND ("Survivorship"[mesh] OR "Survivors"[mesh] OR "Prognosis"[mesh] OR "Survival Analysis"[mesh] OR "Mortality"[mesh] OR "Survival Rate"[mesh] OR (surviv*[tiab] OR prognos*[tiab] OR mortalit*[tiab] OR mortality[sh] OR death*[tiab] OR dead[tiab] OR died[tiab] OR fatal*[tiab] OR outcome*[tiab] OR OS[tiab] OR DFS[tiab] OR PFS[tiab] OR MOS[tiab])) AND ("afghanistan"[mesh] OR "albania"[mesh] OR "algeria"[mesh] OR "american samoa"[mesh] OR "angola"[mesh] OR "argentina"[mesh] OR "armenia"[mesh] OR "azerbaijan"[mesh] OR "bangladesh"[mesh] OR "republic of belarus"[mesh] OR "belize"[mesh] OR "benin"[mesh] OR "bhutan"[mesh] OR "bolivia"[mesh] OR "bosnia and herzegovina"[mesh] OR "botswana"[mesh] OR "brazil"[mesh] OR "bulgaria"[mesh] OR "burkina faso"[mesh] OR "burundi"[mesh] OR "cabo verde"[mesh] OR "cambodia"[mesh] OR "cameroon"[mesh] OR "central african republic"[mesh] OR "chad"[mesh] OR "chile"[mesh] OR "china"[mesh] OR "colombia"[mesh] OR "comoros"[mesh] OR "democratic republic of the congo"[mesh] OR "congo"[mesh] OR "costa rica"[mesh] OR "cote d’ivoire"[mesh] OR "croatia"[mesh] OR "cuba"[mesh] OR "djibouti"[mesh] OR "dominica"[mesh] OR "dominican republic"[mesh] OR "ecuador"[mesh] OR "egypt"[mesh] OR "el salvador"[mesh] OR "equatorial guinea"[mesh] OR "eritrea"[mesh] OR "estonia"[mesh] OR "Eswatini"[Mesh] OR "ethiopia"[mesh] OR "fiji"[mesh] OR "gabon"[mesh] OR "gambia"[mesh] OR "georgia (republic)"[mesh] OR "ghana"[mesh] OR "gibraltar"[mesh] OR "grenada"[mesh] OR "guam"[mesh] OR "guatemala"[mesh] OR "guinea"[mesh] OR "guinea-bissau"[mesh] OR "guyana"[mesh] OR "haiti"[mesh] OR "honduras"[mesh] OR "hungary"[mesh] OR "india"[mesh] OR "indonesia"[mesh] OR "iran"[mesh] OR "iraq"[mesh] OR "jamaica"[mesh] OR "jordan"[mesh] OR "kazakhstan"[mesh] OR "kenya"[mesh] OR "democratic people’s republic of korea"[mesh] OR "kosovo"[mesh] OR "kyrgyzstan"[mesh] OR "laos"[mesh] OR "latvia"[mesh] OR "lebanon"[mesh] OR "lesotho"[mesh] OR "liberia"[mesh] OR "libya"[mesh] OR "lithuania"[mesh] OR "republic of north macedonia"[mesh] OR "madagascar"[mesh] OR "malawi"[mesh] OR "malaysia"[mesh] OR "indian ocean islands"[mesh] OR "mali"[mesh] OR "micronesia"[mesh] OR "palau"[mesh] OR "mauritania"[mesh] OR "mauritius"[mesh] OR "mexico"[mesh] OR "moldova"[mesh] OR "mongolia"[mesh] OR "montenegro"[mesh] OR "morocco"[mesh] OR "mozambique"[mesh] OR "myanmar"[mesh] OR "namibia"[mesh] OR "nepal"[mesh] OR "nicaragua"[mesh] OR "niger"[mesh] OR "nigeria"[mesh] OR "oman"[mesh] OR "pakistan"[mesh] OR "panama"[mesh] OR "papua new guinea"[mesh] OR "paraguay"[mesh] OR "peru"[mesh] OR "philippines"[mesh] OR "poland"[mesh] OR "romania"[mesh] OR "russia"[mesh] OR "rwanda"[mesh] OR "samoa"[mesh] OR "sao tome and principe"[mesh] OR "senegal"[mesh] OR "serbia"[mesh] OR "seychelles"[mesh] OR "sierra leone"[mesh] OR "slovakia"[mesh] OR "slovenia"[mesh] OR "melanesia"[mesh] OR "somalia"[mesh] OR "south africa"[mesh] OR "south sudan"[mesh] OR "sri lanka"[mesh] OR "saint kitts and nevis"[mesh] OR "saint lucia"[mesh] OR "saint vincent and the grenadines"[mesh] OR "sudan"[mesh] OR "suriname"[mesh] OR "syria"[mesh] OR "tajikistan"[mesh] OR "tanzania"[mesh] OR "thailand"[mesh] OR "timor-leste"[mesh] OR "togo"[mesh] OR "tonga"[mesh] OR "trinidad and tobago"[mesh] OR "tunisia"[mesh] OR "turkey"[mesh] OR "turkmenistan"[mesh] OR "uganda"[mesh] OR "ukraine"[mesh] OR "uruguay"[mesh] OR "uzbekistan"[mesh] OR "vanuatu"[mesh] OR "venezuela"[mesh] OR "vietnam"[mesh] OR "middle east"[mesh] OR "yemen"[mesh] OR "yugoslavia"[mesh] OR "zambia"[mesh] OR "zimbabwe"[mesh] OR "africa south of the sahara"[mesh] OR "africa, central"[mesh] OR "africa, northern"[mesh] OR "africa, southern"[mesh] OR "africa, eastern"[mesh] OR "africa, western"[mesh] OR "west indies"[mesh] OR "indian ocean islands"[mesh] OR "caribbean region"[mesh] OR "central america"[mesh] OR "latin america"[mesh] OR "south america"[mesh] OR "asia, central"[mesh] OR "asia, northern"[mesh] OR "asia, southeastern"[mesh] OR "asia, western"[mesh] OR "europe, eastern"[mesh] OR "developing countries"[mesh] OR (afghan*[tiab] OR africa[tiab] OR african[tiab] OR africans[tiab] OR albania[tiab] OR albanian[tiab] OR albanians[tiab] OR algeria[tiab] OR algerian[tiab] OR algerians[tiab] OR angola[tiab] OR angolan[tiab] OR angolans[tiab] OR argentina[tiab] OR argentine[tiab] OR argentines[tiab] OR argentinian[tiab] OR argentinians[tiab] OR armenia[tiab] OR armenian[tiab] OR armenians[tiab] OR azerbaijan[tiab] OR azerbaijani[tiab] OR azerbaijanis[tiab] OR bangladesh[tiab] OR bangladeshi[tiab] OR bangladeshis[tiab] OR bangalee[tiab] OR bajan[tiab] OR belarus*[tiab] OR byelarus*[tiab] OR belorus*[tiab] OR byelorus*[tiab] OR belize[tiab] OR belizean[tiab] OR benin[tiab] OR beninese[tiab] OR benineses[tiab] OR dahomey[tiab] OR bhutan[tiab] OR bhutanese[tiab] OR bolivia[tiab] OR bolivian[tiab] OR bolivians[tiab] OR bosnia[tiab] OR bosnian[tiab] OR bosnians[tiab] OR herzegovina[tiab] OR herzegovinian[tiab] OR herzegovinians[tiab] OR botswana[tiab] OR botswanan[tiab] OR botswanans[tiab] OR batswana[tiab] OR bechuanaland[tiab] OR brazil[tiab] OR brazilian[tiab] OR brazilians[tiab] OR brasil[tiab] OR brasilian[tiab] OR brasilians[tiab] OR bulgaria[tiab] OR bulgarian[tiab] OR bulgarians[tiab] OR burkina[tiab] OR burkinabe[tiab] OR burkinabes[tiab] OR upper-volta[tiab] OR burundi[tiab] OR burundian[tiab] OR urundi[tiab] OR cabo-verde[tiab] OR cape-verde[tiab] OR cape-verdean[tiab] OR cape-verdeans[tiab] OR cambodia[tiab] OR cambodian[tiab] OR cambodians[tiab] OR kampuchea[tiab] OR kampuchean[tiab] OR kampucheans[tiab] OR khmer[tiab] OR khmers[tiab] OR cameroon[tiab] OR cameroons[tiab] OR cameroonian[tiab] OR cameroonians[tiab] OR cameroonese[tiab] OR cameron[tiab] OR cameronian[tiab] OR cameronians[tiab] OR cameroun[tiab] OR ubangi[tiab] OR chad[tiab] OR chadian[tiab] OR chadians[tiab] OR chile[tiab] OR chilean[tiab] OR chileans[tiab] OR china[tiab] OR chinese[tiab] OR colombia[tiab] OR colombian[tiab] OR colombians[tiab] OR colombiano[tiab] OR colombianos[tiab] OR comoro[tiab] OR comoros[tiab] OR mayotte[tiab] OR congo[tiab] OR congolese[tiab] OR zaire[tiab] OR zairean[tiab] OR zaireans[tiab] OR zairese[tiab] OR costa-rica[tiab] OR costa-rican[tiab] OR costa-ricans[tiab] OR cote-d-ivoire[tiab] OR ivory-coast[tiab] OR ivorian[tiab] OR ivorians[tiab] OR croatia[tiab] OR croatian[tiab] OR croatians[tiab] OR cuba[tiab] OR cuban[tiab] OR cubans[tiab] OR djibouti[tiab] OR djiboutian[tiab] OR djiboutians[tiab] OR dominica[tiab] OR dominican[tiab] OR dominicans[tiab] OR ecuador[tiab] OR ecuadorian[tiab] OR ecuadorians[tiab] OR egypt[tiab] OR egyptian[tiab] OR egyptians[tiab] OR united-arab-republic[tiab] OR salvador[tiab] OR salvadoran[tiab] OR salvadorans[tiab] OR equatoguinean[tiab] OR equatoguineans[tiab] OR eritrea[tiab] OR eritrean[tiab] OR eritreans[tiab] OR estonia[tiab] OR estonian[tiab] OR estonians[tiab] OR eswatini[tiab] OR swaziland[tiab] OR swazi[tiab] OR swazis[tiab] OR swati[tiab] OR ethiopia[tiab] OR ethiopian[tiab] OR ethiopians[tiab] OR fiji[tiab] OR fijian[tiab] OR fijians[tiab] OR gabon[tiab] OR gabonese[tiab] OR gambia[tiab] OR gambian[tiab] OR gambians[tiab] OR georgia[tiab] OR georgian[tiab] OR georgians[tiab] OR ghana[tiab] OR ghanaian[tiab] OR ghanaians[tiab] OR gold-coast[tiab] OR gibraltar[tiab] OR gibraltarian[tiab] OR gibraltarians[tiab] OR grenada[tiab] OR grenadian[tiab] OR grenadians[tiab] OR guam[tiab] OR guamanian[tiab] OR guamanians[tiab] OR guatemala[tiab] OR guatemalan[tiab] OR guatemalans[tiab] OR guinea[tiab] OR guinean[tiab] OR guineans[tiab] OR guyana[tiab] OR guyanese[tiab] OR guiana[tiab] OR guianan[tiab] OR haiti[tiab] OR haitian[tiab] OR haitians[tiab] OR hispaniola[tiab] OR honduras[tiab] OR honduran[tiab] OR hondurans[tiab] OR hungary[tiab] OR hungarian[tiab] OR hungarians[tiab] OR india[tiab] OR indian[tiab] OR indians[tiab] OR indonesia[tiab] OR indonesia[tiab] OR indonesia[tiab] OR iran[tiab] OR irani[tiab] OR iranis[tiab] OR iranian[tiab] OR iranians[tiab] OR iraq[tiab] OR iraqi[tiab] OR iraqis[tiab] OR iraqian[tiab] OR jamaica[tiab] OR jamaican[tiab] OR jamaicans[tiab] OR jordan[tiab] OR jordanian[tiab] OR jordanians[tiab] OR kazak*[tiab] OR kenya[tiab] OR kenyan[tiab] OR kenyans[tiab] OR north-korea[tiab] OR north-korean[tiab] OR north-koreans[tiab] OR democratic-people-s-republic-of-korea[tiab] OR kosov*[tiab] OR kirgiz*[tiab] OR kyrgyz*[tiab] OR kirghiz*[tiab] OR lao[tiab] OR laos[tiab] OR loatian*[tiab] OR latvia[tiab] OR latvian[tiab] OR latvians[tiab] OR lebanon[tiab] OR lebanese[tiab] OR lesotho[tiab] OR basutoland[tiab] OR mosotho[tiab] OR basotho[tiab] OR liberia[tiab] OR liberian[tiab] OR liberians[tiab] OR libya[tiab] OR libyan[tiab] OR libyans[tiab] OR lithuania[tiab] OR lithuanian[tiab] OR lithuanians[tiab] OR macedonia[tiab] OR macedonian[tiab] OR macedonians[tiab] OR madagascar[tiab] OR madagascan[tiab] OR madagascans[tiab] OR malagasy[tiab] OR malawi[tiab] OR malawian[tiab] OR malawians[tiab] OR nyasaland[tiab] OR malaysia*[tiab] OR maldiv*[tiab] OR mali[tiab] OR malian*[tiab] OR malians[tiab] OR micronesia*[tiab] OR kiribati[tiab] OR marshall-island*[tiab] OR marshallese[tiab] OR mariana[tiab] OR marianas[tiab] OR palau[tiab] OR palauan[tiab] OR palauans[tiab] OR tuvalu[tiab] OR tuvaluan[tiab] OR tuvaluans[tiab] OR mauritania[tiab] OR mauritanian[tiab] OR mauritanians[tiab] OR mauritius[tiab] OR mauritian[tiab] OR mauritians[tiab] OR mexico[tiab] OR mexican[tiab] OR mexicans[tiab] OR moldova[tiab] OR moldovan[tiab] OR moldovans[tiab] OR mongolia[tiab] OR mongolian[tiab] OR mongolians[tiab] OR mongol[tiab] OR mongols[tiab] OR montenegro[tiab] OR montenegrin[tiab] OR montenegrins[tiab] OR morocco[tiab] OR moroccan[tiab] OR moroccans[tiab] OR mozambique[tiab] OR mozambican[tiab] OR mozambicans[tiab] OR myanmar[tiab] OR myanma[tiab] OR myanmarese[tiab] OR burma[tiab] OR burmese[tiab] OR namibia[tiab] OR namibian[tiab] OR namibians[tiab] OR nauru[tiab] OR nauruan[tiab] OR nauruans[tiab] OR nepal[tiab] OR nepali[tiab] OR nepalis[tiab] OR nepalese[tiab] OR nicaragua[tiab] OR nicaraguan[tiab] OR nicaraguans[tiab] OR niger[tiab] OR nigeria[tiab] OR nigerian[tiab] OR nigerians[tiab] OR nigerien[tiab] OR nigeriens[tiab] OR oman[tiab] OR omani[tiab] OR omanis[tiab] OR muscat[tiab] OR pakistan[tiab] OR pakistani[tiab] OR pakistanis[tiab] OR panama[tiab] OR panamanian[tiab] OR panamanians[tiab] OR paraguay[tiab] OR paraguayan[tiab] OR paraguayans[tiab] OR peru[tiab] OR peruvian[tiab] OR peruvians[tiab] OR philippin*[tiab] OR philipin*[tiab] OR phillipin*[tiab] OR phillippin*[tiab] OR filipin*[tiab] OR poland[tiab] OR polish[tiab] OR pole[tiab] OR poles[tiab] OR romania[tiab] OR romanian[tiab] OR romanians[tiab] OR russia[tiab] OR russian[tiab] OR russians[tiab] OR ussr[tiab] OR soviet[tiab] OR soviets[tiab] OR rwand*[tiab] OR ruand*[tiab] OR samoa[tiab] OR samoan[tiab] OR samoans[tiab] OR pacific-island[tiab] OR pacific-islands[tiab] OR pacific-islander[tiab] OR pacific-islanders[tiab] OR polynesia[tiab] OR polynesian[tiab] OR polynesians[tiab] OR sao-tome*[tiab] OR senegal[tiab] OR senegalese[tiab] OR serbia[tiab] OR serbian[tiab] OR serbians[tiab] OR seychelles[tiab] OR seychellois[tiab] OR seychelloise[tiab] OR sierra-leone[tiab] OR sierra-leonean[tiab] OR sierra-leoneans[tiab] OR slovak[tiab] OR slovaks[tiab] OR slovakia[tiab] OR slovakian[tiab] OR slovakians[tiab] OR slovenia[tiab] OR slovenian[tiab] OR slovenians[tiab] OR slovene[tiab] OR slovenes[tiab] OR melanesia[tiab] OR melanesian[tiab] OR melanesians[tiab] OR solomon-island[tiab] OR solomon-islands[tiab] OR solomon-islander[tiab] OR solomon-islanders[tiab] OR norfolk-island*[tiab] OR somali[tiab] OR somalian[tiab] OR somalians[tiab] OR sri-lanka[tiab] OR sri-lankan[tiab] OR sri-lankans[tiab] OR ceylon[tiab] OR ceylonese[tiab] OR saint-kitts[tiab] OR st-kitts[tiab] OR kittitian[tiab] OR kittitians[tiab] OR saint-lucia[tiab] OR saint-lucian[tiab] OR st-lucia[tiab] OR saint-vincent[tiab] OR st-vincent[tiab] OR vincentian[tiab] OR vincentians[tiab] OR grenadines[tiab] OR sudan[tiab] OR sudanese[tiab] OR surinam[tiab] OR suriname[tiab] OR surinamese[tiab] OR syria[tiab] OR syrian[tiab] OR syrians[tiab] OR tadjikistan[tiab] OR tadzhikistan[tiab] OR tadzhik[tiab] OR tajikistan[tiab] OR tajik[tiab] OR tajikistani[tiab] OR tanzania[tiab] OR tanzanian[tiab] OR tanzanians[tiab] OR tanganyikan[tiab] OR thailand[tiab] OR thai[tiab] OR thais[tiab] OR siam[tiab] OR timor[tiab] OR timorese[tiab] OR timoreses[tiab] OR togo[tiab] OR togolese[tiab] OR tonga[tiab] OR tongan[tiab] OR tongans[tiab] OR trinidad[tiab] OR trinidadian[tiab] OR trinidadians[tiab] OR tobagonian[tiab] OR tobagonians[tiab] OR tunisia[tiab] OR tunisian[tiab] OR tunisians[tiab] OR turkey[tiab] OR turk[tiab] OR turks[tiab] OR turkish[tiab] OR turkmenistan[tiab] OR turkmen[tiab] OR turkmens[tiab] OR uganda[tiab] OR ugandan[tiab] OR ugandans[tiab] OR ukraine[tiab] OR ukrainian[tiab] OR ukrainians[tiab] OR uruguay[tiab] OR uruguayan[tiab] OR uruguayans[tiab] OR uzbekistan[tiab] OR uzbek[tiab] OR uzbeks[tiab] OR vanuatu[tiab] vanuatuan[tiab] OR vanuatuans[tiab] OR new-hebrides[tiab] OR venezuela[tiab] OR venezuelan[tiab] OR venezuelans[tiab] OR vietnam[tiab] OR vietnamese[tiab] OR viet-nam[tiab] OR viet-namese[tiab] OR middle-east[tiab] OR middle-eastern[tiab] OR west-bank[tiab] OR gaza[tiab] OR palestine[tiab] OR palestinian[tiab] OR palestinians[tiab] OR yemen*[tiab] OR yugoslav*[tiab] OR zambia[tiab] OR zambian[tiab] OR zambians[tiab] OR zimbabwe[tiab] OR zimbabwean[tiab] OR zimbabweans[tiab] OR rhodesia[tiab] OR rhodesian[tiab] OR rhodesians[tiab] OR global-south[tiab] OR maghreb[tiab] OR maghrib[tiab] OR sahara*[tiab] OR west-indies[tiab] OR caribbean[tiab] OR caribbeans[tiab] OR central-america[tiab] OR central-american[tiab] OR central-americans[tiab] OR latin-america[tiab] OR latin-american[tiab] OR latin-americans[tiab] OR south-america[tiab] OR south-american[tiab] OR south-americans[tiab] OR central-asia[tiab] OR central-asian[tiab] OR central-asians[tiab] OR north-asia[tiab] OR north-asian[tiab] OR north-asians[tiab] OR northern-asia[tiab] OR northern-asian[tiab] OR northern-asians[tiab] OR southeastern-asia[tiab] OR southeastern-asian[tiab] OR southeastern-asians[tiab] OR south-eastern-asia[tiab] OR south-eastern-asian[tiab] OR southeast-asia[tiab] OR southeast-asian[tiab]OR southeast-asians[tiab]OR south-east-asia[tiab] OR south-east-asian[tiab] OR south-east-asians[tiab] OR west-asia[tiab] OR west-asian[tiab] OR west-asians[tiab] OR western-asia[tiab] OR western-asian[tiab] OR western-asians[tiab] OR east-europe[tiab] OR east-european[tiab] OR east-europeans[tiab] OR eastern-europe[tiab] OR eastern-european[tiab] OR eastern-europeans[tiab] OR afghan*[ad] OR africa[ad] OR african[ad] OR albania[ad] OR albanian[ad] OR algeria[ad] OR algerian[ad] OR angola[ad] OR angolan[ad] OR argentina[ad] OR argentine[ad] OR argentinian[ad] OR armenia[ad] OR armenian[ad] OR azerbaijan[ad] OR azerbaijani[ad] OR bangladesh[ad] OR bangladeshi[ad] OR belarus*[ad] OR byelarus*[ad] OR belorus*[ad] OR byelorus*[ad] OR belize[ad] OR benin[ad] OR beninese[ad] OR dahomey[ad] OR bhutan[ad] OR bhutanese[ad] OR bolivia[ad] OR bolivian[ad] OR bosnia[ad] OR bosnian[ad] OR herzegovina[ad] OR herzegovinian[ad] OR botswana[ad] OR brazil[ad] OR brazilian[ad] OR brasil[ad] OR brasilian[ad] OR bulgaria[ad] OR bulgarian[ad] OR burkina[ad] OR burkinabe[ad] OR burundi[ad] OR cabo-verde[ad] OR cape-verde[ad] OR cambodia[ad] OR cambodian[ad] OR kampuchea[ad] OR khmer[ad] OR cameroon[ad] OR cameron[ad] OR cameroun[ad] OR chad[ad] OR chadian[ad] OR chile[ad] OR chilean[ad] OR china[ad] OR chinese[ad] OR colombia[ad] OR colombian[ad] OR comoro[ad] OR comoros[ad] OR mayotte[ad] OR congo[ad] OR congolese[ad] OR zaire[ad] OR zairean[ad] OR costa-rica[ad] OR costa-rican[ad] OR cote-d-ivoire[ad] OR ivory-coast[ad] OR ivorian[ad] OR croatia[ad] OR croatian[ad] OR cuba[ad] OR cuban[ad] OR djibouti[ad] OR djiboutian[ad] OR dominica[ad] OR dominican[ad] OR ecuador[ad] OR ecuadorian[ad] OR egypt[ad] OR egyptian[ad] OR united-arab-republic[ad] OR salvador[ad] OR salvadoran[ad] OR eritrea[ad] OR eritrean[ad] OR estonia[ad] OR eswatini[ad] OR swaziland[ad] OR ethiopia[ad] OR ethiopian[ad] OR fiji[ad] OR fijian[ad] OR gabon[ad] OR gambia[ad] OR gambian[ad] OR georgia[ad] OR georgian[ad] OR ghana[ad] OR ghanaian[ad] OR gibraltar[ad] OR grenada[ad] OR guam[ad] OR guatemala[ad] OR guatemalan[ad] OR guinea[ad] OR guinean[ad] OR guyana[ad] OR guiana[ad] OR guianan[ad] OR haiti[ad] OR haitian[ad] OR hispaniola[ad] OR honduras[ad] OR honduran[ad] OR hungary[ad] OR hungarian[ad] OR india[ad] OR indian[ad] OR indians[ad] OR indonesia[ad] OR indonesian[ad] OR iran[ad] OR iranian[ad] OR iraq[ad] OR iraqi[ad] OR jamaica[ad] OR jamaican[ad] OR jordan[ad] OR jordanian[ad] OR kazakhstan[ad] OR kazakstan[ad] OR kenya[ad] OR kenyan[ad] OR north-korea[ad] OR north-korean[ad] OR kosov*[ad] OR kirgiz*[ad] OR kyrgyz*[ad] OR kirghiz*[ad] OR lao[ad] OR laos[ad] OR latvia[ad] OR latvian[ad] OR lebanon[ad] OR lebanese[ad] OR lesotho[ad] OR basotho[ad] OR liberia[ad] OR liberian[ad] OR libya[ad] OR libyan[ad] OR lithuania[ad] OR lithuanian[ad] OR macedonia[ad] OR macedonian[ad] OR madagascar[ad] OR malagasy[ad] OR malawi[ad] OR malawian[ad] OR malaysia*[ad] OR maldiv*[ad] OR mali[ad] OR malian[ad] OR malians[ad] OR micronesia*[ad] OR kiribati[ad] OR marshall-islands[ad] OR marshallese[ad] OR nauru[ad] OR mariana[ad] OR marianas[ad] OR palau[ad] OR tuvalu[ad] OR mauritania[ad] OR mauritanian[ad] OR mauritius[ad] OR mauritian[ad] OR mexico[ad] OR mexican[ad] OR moldova[ad] OR moldovan[ad] OR mongolia[ad] OR mongolian[ad] OR mongol[ad] OR montenegro[ad] OR montenegrin[ad] OR morocco[ad] OR moroccan[ad] OR mozambique[ad] OR mozambican[ad] OR myanmar[ad] OR myanma[ad] OR burma[ad] OR burmese[ad] OR burmese[ad] OR namibia[ad] OR namibian[ad] OR nepal[ad] OR nepalese[ad] OR nicaragua[ad] OR nicaraguan[ad] OR niger[ad] OR nigeria[ad] OR nigerian[ad] OR oman[ad] OR omani[ad] OR muscat[ad] OR pakistan[ad] OR pakistani[ad] OR panama[ad] OR panamanian[ad] OR paraguay[ad] OR paraguayan[ad] OR peru[ad] OR peruvian*[ad] OR philippin*[ad] OR philipin*[ad] OR phillipin*[ad] OR phillippin*[ad] OR filipin*[ad] OR poland[ad] OR polish[ad] OR rhodesia[ad] OR rhodesian[ad] OR romania[ad] OR romanian[ad] OR russia[ad] OR russian[ad] OR ussr[ad] OR soviet[ad] OR rwand*[ad] OR ruand*[ad] OR samoa[ad] OR samoan[ad] OR pacific-island[ad] OR pacific-islands[ad] OR pacific-islander[ad] OR pacific-islanders[ad] OR polynesia[ad] OR polynesian[ad] OR sao-tome-and-principe[ad] OR sao-tome[ad] OR senegal[ad] OR senegalese[ad] OR serbia[ad] OR serbian[ad] OR seychelles[ad] OR sierra-leone[ad] OR slovak[ad] OR slovakia[ad] OR slovakian[ad] OR slovenia[ad] OR slovenian[ad] OR slovene[ad] OR melanesia[ad] OR melanesian[ad] OR solomon-island[ad] OR solomon-islands[ad] OR norfolk-island[ad] OR somalia[ad] OR sri-lanka[ad] OR sri-lankan[ad] OR ceylon[ad] OR saint-kitts[ad] OR st-kitts[ad] OR saint-lucia[ad] OR st-lucia[ad] OR saint-vincent[ad] OR st-vincent[ad] OR grenadines[ad] OR sudan[ad] OR sudanese[ad] OR surinam[ad] OR suriname[ad] OR syria[ad] OR syrian[ad] OR tadjikistan[ad] OR tadzhikistan[ad] OR tadzhik[ad] OR tajikistan[ad] OR tajik[ad] OR tanzania[ad] OR tanzanian[ad] OR thailand[ad] OR thai[ad] OR siam[ad] OR timor[ad] OR togo[ad] OR togolese[ad] OR tonga[ad] OR tongan[ad] OR trinidad[ad] OR tobago[ad] OR tunisia[ad] OR tunisian[ad] OR turkey[ad] OR turk[ad] OR turks[ad] OR turkish[ad] OR turkmenistan[ad] OR turkmen[ad] OR uganda[ad] OR ugandan[ad] OR ukraine[ad] OR ukrainian[ad] OR uruguay[ad] OR uruguayan[ad] OR uzbekistan[ad] OR uzbek[ad] OR vanuatu[ad] OR venezuela[ad] OR venezuelan[ad] OR vietnam[ad] OR vietnamese[ad] OR viet-nam[ad] OR viet-namese[ad] OR middle-east[ad] OR middle-eastern[ad] OR west-bank[ad] OR gaza[ad] OR palestine[ad] OR palestinian[ad] OR yemen[ad] OR yemeni[ad] OR yugoslav*[ad] OR zambia[ad] OR zambian[ad] OR zimbabwe[ad] OR global-south[ad] OR sahara*[ad] OR west-indies[ad] OR caribbean[ad] OR central-america*[ad] OR central-american[ad] OR latin-america[ad] OR latin-american[ad] OR south-america[ad] OR south-american[ad] OR central-asia[ad] OR central-asian[ad] OR north-asia[ad] OR northern-asia[ad] OR southeastern-asia[ad] OR southeast-asia[ad] OR southeast-asian[ad] OR south-east-asia[ad] OR south-east-asian[ad] OR west-asia[ad] OR west-asian[ad] OR western-asia[ad] OR east-europe[ad] OR east-european[ad] OR eastern-europe[ad] OR eastern-european[ad] OR lmic[tiab] OR lmics[tiab] OR third-world[tiab] OR lami-countr*[tiab] OR transitional-countr*[tiab] OR ((developing[tiab] OR less-developed[tiab] OR lesser-developed[tiab] OR under-developed[tiab] OR underdeveloped[tiab] OR middle-income[tiab] OR low-income[tiab] OR lower-income[tiab] OR underserved[tiab] OR under-served[tiab] OR deprived[tiab] OR poor[tiab] OR poorer[tiab] OR improverished[tiab]) AND (country[tiab] OR countries[tiab] OR nation[tiab] OR nations[tiab] OR population[tiab] OR populations[tiab] OR world*[tiab] OR economy[tiab] OR economies[tiab])) OR ((low[tiab] OR lower[tiab]) AND (gdp[tiab] OR gnp[tiab] OR gross-domestic[tiab] OR gross-national[tiab])) OR ((emerging[tiab]) AND (econom*[tiab] OR nation*[tiab])))) NOT ("Animals"[Mesh] NOT ("Animals"[Mesh] AND "Humans"[Mesh])) NOT ("Editorial"[pt] OR "Comment"[pt] OR “Clinical Trial Protocol”[pt] OR case-report*[ti] OR systematic-review*[ti] OR meta-analys*[ti]) AND (2005/01/01:3000[pdat])

**---**

**Web of Science Core Collection (Clarivate):
7097 Results**
(TS=(((large-intestine* OR large-bowel* OR cecum* OR c?ecum* OR colorect* OR colon* OR rectum* OR rectal OR sigmoid* OR abdominoperineal*) NEAR/3 (neoplas* OR cancer* OR adenoma* OR carcinoma* OR adenocancer* OR adenocarcinoma* OR malignan* OR tumor* OR tumour* OR metasta* OR lesion* OR mass*) )) OR TS=(((perineal*) AND (abdomen OR abdomin*) ) AND (neoplas* OR cancer* OR adenoma* OR carcinoma* OR adenocancer* OR adenocarcinoma* OR malignan* OR tumor* OR tumour* OR metasta* OR lesion* OR mass*) ) OR TS=(((flexur*) AND (colon* OR spleen* OR splenic OR hepatic*) ) AND (neoplas* OR cancer* OR adenoma* OR carcinoma* OR adenocancer* OR adenocarcinoma* OR malignan* OR tumor* OR tumour* OR metasta* OR lesion* OR mass*))) AND (TS=(colectom* OR hemicolectom* OR proctectom* OR proctocolectom* OR coloproctectom* OR surger* OR surgical* OR resection* OR debulk* OR excision*)) AND (TS=(surviv* OR prognos* OR mortalit* OR death* OR dead OR died OR fatal* OR outcome* OR OS OR DFS OR PFS OR MOS)) AND ((TS=(afghan* OR africa* OR albania* OR algeria* OR angola* OR argentin* OR armenia* OR azerbaijan* OR bangladesh* OR bangalee* OR bajan* OR belarus* OR byelarus* OR belorus* OR byelorus* OR belize* OR benin* OR dahomey OR bhutan* OR bolivia* OR bosnia* OR herzegovin* OR botswana* OR batswana* OR bechuanaland* OR brazil* OR brasil* OR bulgaria* OR burkina* OR upper-volta OR burundi* OR urundi* OR cabo-verde* OR cape-verde* OR cambodia* OR kampuchea* OR khmer* OR cameroon* OR cameron* OR cameroun* OR ubangi* OR chad OR chadian* OR chile OR chilean* OR china OR chinese OR colombia* OR comoro* OR comorian* OR mayotte* OR congo OR congolese OR zaire* OR costa-rica* OR cote-d-ivoir* OR ivory-coast* OR ivorian* OR croatia* OR cuba* OR djibouti* OR dominica* OR ecuador* OR egypt* OR united-arab-republic* OR salvador* OR equatoguinean* OR eritrea* OR estonia* OR eswatin* OR swazi* OR swati* OR ethiopia* OR fiji* OR gabon* OR gambia* OR georgia* OR ghana* OR gold-coast* OR gibraltar* OR grenad* OR guam* OR guatemala* OR guinea* OR guyan* OR guiana* OR haiti* OR hispaniola OR hondura* OR hungar* OR india* OR indonesia* OR timor* OR iran* OR iraq* OR jamaica* OR jordan* OR kazakh* OR kenya* OR north-korea* OR kosov* OR kyrgyz* OR kirghiz* OR kirgiz* OR kirghiz* OR lao OR laos OR loatian* OR latvia* OR lebanon OR lebanese OR lesoth* OR basutoland OR mosotho* OR basotho* OR liberia* OR libya* OR lithuania* OR macedonia* OR madagasca* OR malagasy* OR malawi* OR nyasaland OR malaysia* OR maldiv* OR mali OR malian OR malians OR micronesia* OR kiribati* OR marshall-island* OR marshallese OR nauru* OR mariana* OR palau* OR tuvalu* OR mauritania* OR mauritian* OR mauritius OR mexico OR mexican* OR moldov* OR mongol* OR montenegr* OR morocc* OR ifni OR mozambi* OR myanma* OR burma* OR burmese* OR namibia* OR nauruan* OR nepal* OR nicaragua* OR niger OR nigeria* OR nigerien* OR oman* OR muscat OR pakistan* OR panama* OR paraguay* OR peru OR peruvian* OR philippin* OR philipin* OR phillipin* OR phillippin* OR filipin* OR poland OR polish OR pole OR poles OR romania* OR russia* OR ussr OR soviet-union* OR union-of-soviet-socialist-republic* OR rwand* OR ruand* OR samoa* OR pacific-island* OR polynesia* OR sao-tome* OR senegal* OR serbia* OR seychell* OR sierra-leone* OR slovak* OR melanesia* OR solomon-island* OR norfolk-island* OR somali* OR sri-lanka* OR ceylon* OR saint-kitt* OR st-kitt* OR kittitian* OR saint-lucia* OR st-lucia* OR saint-vincent* OR st-vincent* OR vincentian* OR grenadin* OR sudan* OR surinam* OR syria* OR tadjik* OR tadzhik* OR tajik* OR tanzania* OR tanganyika* OR thailand* OR thai OR thais OR siam OR timor* OR togo OR togolese* OR tonga* OR trinidad* OR tobago* OR tunisia* OR turkey OR turk OR turks OR turkmen* OR uganda* OR ukrain* OR uruguay* OR uzbek* OR vanuatu* OR new-hebride* OR venezuela* OR vietnam* OR viet-nam OR viet-names* OR middle-east* OR west-bank OR gaza OR palestin* OR yemen* OR yugoslav* OR zambia* OR zimbabwe* OR rhodesia* OR global-south OR maghreb* OR maghrib* OR sahara* OR west-indies OR caribbean* OR central-america* OR latin-america* OR south-america* OR central-asia* OR north-asia* OR northern-asia* OR southeastern-asia* OR south-eastern-asia* OR southeast-asia* OR south-east-asia* OR west-asia* OR western-asia* OR east-europe* OR eastern-europe* OR lmic OR lmics OR third-world OR lami-countr* OR transitional-countr* OR ((developing OR less-developed OR lesser-developed OR under-developed OR underdeveloped OR middle-income OR low-income OR lower-income OR underserved OR under-served OR deprived OR poor OR poorer) NEAR/3 (countr* OR nation* OR population* OR world* OR econom*)) OR ((low OR lower) NEAR/3 (gdp OR gnp OR gross-domestic OR gross-national)) OR ((emerging) NEAR/3 (econom* OR nation*)))) OR (CU=(afghan* OR africa* OR albania* OR algeria* OR angola* OR argentin* OR armenia* OR azerbaijan* OR bangladesh* OR bangalee* OR bajan* OR belarus* OR byelarus* OR belorus* OR byelorus* OR belize* OR benin* OR dahomey OR bhutan* OR bolivia* OR bosnia* OR herzegovin* OR botswana* OR batswana* OR bechuanaland* OR brazil* OR brasil* OR bulgaria* OR burkina* OR upper-volta OR burundi* OR urundi* OR cabo-verde* OR cape-verde* OR cambodia* OR kampuchea* OR khmer* OR cameroon* OR cameron* OR cameroun* OR ubangi* OR chad OR chadian* OR chile OR chilean* OR china OR chinese OR colombia* OR comoro* OR comorian* OR mayotte* OR congo OR congolese OR zaire* OR costa-rica* OR cote-d-ivoir* OR ivory-coast* OR ivorian* OR croatia* OR cuba* OR djibouti* OR dominica* OR ecuador* OR egypt* OR united-arab-republic* OR salvador* OR equatoguinean* OR eritrea* OR estonia* OR eswatin* OR swazi* OR swati* OR ethiopia* OR fiji* OR gabon* OR gambia* OR georgia* OR ghana* OR gold-coast* OR gibraltar* OR grenad* OR guam* OR guatemala* OR guinea* OR guyan* OR guiana* OR haiti* OR hispaniola OR hondura* OR hungar* OR india* OR indonesia* OR timor* OR iran* OR iraq* OR jamaica* OR jordan* OR kazakh* OR kenya* OR north-korea* OR kosov* OR kyrgyz* OR kirghiz* OR kirgiz* OR kirghiz* OR lao OR laos OR loatian* OR latvia* OR lebanon OR lebanese OR lesoth* OR basutoland OR mosotho* OR basotho* OR liberia* OR libya* OR lithuania* OR macedonia* OR madagasca* OR malagasy* OR malawi* OR nyasaland OR malaysia* OR maldiv* OR mali OR malian OR malians OR micronesia* OR kiribati* OR marshall-island* OR marshallese OR nauru* OR mariana* OR palau* OR tuvalu* OR mauritania* OR mauritian* OR mauritius OR mexico OR mexican* OR moldov* OR mongol* OR montenegr* OR morocc* OR ifni OR mozambi* OR myanma* OR burma* OR burmese* OR namibia* OR nauruan* OR nepal* OR nicaragua* OR niger OR nigeria* OR nigerien* OR oman* OR muscat OR pakistan* OR panama* OR paraguay* OR peru OR peruvian* OR philippin* OR philipin* OR phillipin* OR phillippin* OR filipin* OR poland OR polish OR pole OR poles OR romania* OR russia* OR ussr OR soviet-union* OR union-of-soviet-socialist-republic* OR rwand* OR ruand* OR samoa* OR pacific-island* OR polynesia* OR sao-tome* OR senegal* OR serbia* OR seychell* OR sierra-leone* OR slovak* OR melanesia* OR solomon-island* OR norfolk-island* OR somali* OR sri-lanka* OR ceylon* OR saint-kitt* OR st-kitt* OR kittitian* OR saint-lucia* OR st-lucia* OR saint-vincent* OR st-vincent* OR vincentian* OR grenadin* OR sudan* OR surinam* OR syria* OR tadjik* OR tadzhik* OR tajik* OR tanzania* OR tanganyika* OR thailand* OR thai OR siam OR timor* OR togo OR togolese* OR tonga* OR trinidad* OR tobago* OR tunisia* OR turkey OR turk OR turks OR turkmen* OR uganda* OR ukrain* OR uruguay* OR uzbek* OR vanuatu* OR new-hebride* OR venezuela* OR vietnam* OR viet-nam OR viet-names* OR middle-east* OR west-bank OR gaza OR palestin* OR yemen* OR yugoslav* OR zambia* OR zimbabwe* OR rhodesia* OR global-south OR magreb* OR maghrib* OR sahara* OR west-indies OR caribbean* OR central-america* OR latin-america* OR south-america* OR central-asia* OR north-asia* OR northern-asia* OR southeastern-asia* OR south-eastern-asia* OR southeast-asia* OR south-east-asia* OR west-asia* OR western-asia* OR east-europe* OR eastern-europe*))) AND (PY=(2005-2021)) NOT (TS=((animal* OR nonhuman* OR rat OR rats OR mouse OR mice OR rodent* OR murine* OR primate* OR monkey* OR dog OR dogs OR canine* OR pig* OR porcine*) NOT (human* AND (animal* OR nonhuman* OR rat OR rats OR mouse OR mice OR rodent* OR murine* OR primate* OR monkey* OR dog OR dogs OR canine* OR pig* OR porcine*)))) NOT (TI=(editorial* OR comment* OR case-report* OR systematic-review* OR meta-analys* OR ((trial*) AND (protocol*)))) AND

**Embase (Scopus)
7918 Results**

(TITLE-ABS-KEY(large-intestine* OR large-bowel* OR cecum* OR c?ecum* OR colorect* OR colon* OR rectum* OR rectal OR sigmoid* OR abdominoperineal* OR ((flexur*) AND (colon* OR spleen* OR splenic OR hepatic*)) OR ((abdomen OR abdomin*) AND (perineal*)) W/3 (neoplas* OR cancer* OR adenoma* OR carcinoma* OR adenocancer* OR adenocarcinoma* OR malignan* OR tumor* OR tumour* OR metasta* OR lesion* OR mass*)) AND TITLE-ABS-KEY(colectom* OR hemicolectom* OR proctectom* OR proctocolectom* OR coloproctectom* OR surger* OR surgical* OR resection* OR debulk* OR excision*)) AND TITLE-ABS-KEY(surviv* OR prognos* OR mortalit* OR death* OR dead OR died OR fatal* OR outcome* OR {OS} OR {DFS} OR {PFS} OR {MOS}) AND ((TITLE-ABS-KEY(afghan* OR africa* OR albania* OR algeria* OR angola* OR argentin* OR armenia* OR azerbaijan* OR bangladesh* OR bangalee* OR bajan* OR belarus* OR byelarus* OR belorus* OR byelorus* OR belize* OR benin* OR dahomey OR bhutan* OR bolivia* OR bosnia* OR herzegovin* OR botswana* OR batswana* OR bechuanaland* OR brazil* OR brasil* OR bulgaria* OR burkina* OR upper-volta OR burundi* OR urundi* OR cabo-verde* OR cape-verde* OR cambodia* OR kampuchea* OR khmer* OR cameroon* OR cameron* OR cameroun* OR ubangi* OR chad OR chadian* OR chile OR chilean* OR china OR chinese OR colombia* OR comoro* OR comorian* OR mayotte* OR congo OR congolese OR zaire* OR costa-rica* OR cote-d-ivoir* OR ivory-coast* OR ivorian* OR croatia* OR cuba* OR djibouti* OR dominica* OR ecuador* OR egypt* OR united-arab-republic* OR salvador* OR equatoguinean* OR eritrea* OR estonia* OR eswatin* OR swazi* OR swati* OR ethiopia* OR fiji* OR gabon* OR gambia* OR georgia* OR ghana* OR gold-coast* OR gibraltar* OR grenad* OR guam* OR guatemala* OR guinea* OR guyan* OR guiana* OR haiti* OR hispaniola OR hondura* OR hungar* OR india* OR indonesia* OR timor* OR iran* OR iraq* OR jamaica* OR jordan* OR kazakh* OR kenya* OR north-korea* OR kosov* OR kyrgyz* OR kirghiz* OR kirgiz* OR kirghiz* OR lao OR laos OR loatian* OR latvia* OR lebanon* lebanese OR lesoth* OR basutoland OR mosotho* OR basotho* OR liberia* OR libya* OR lithuania* OR macedonia* OR madagasca* OR malagasy* OR malawi* OR nyasaland OR malaysia* OR maldiv* OR mali OR malian OR malians OR micronesia* OR kiribati* OR marshall-island* OR marshallese OR nauru* OR mariana* OR palau* OR tuvalu* OR mauritania* OR mauritian* OR mauritius OR mexico OR mexican* OR moldov* OR mongol* OR montenegr* OR morocc* OR ifni OR mozambi* OR myanma* OR burma* OR burmese* OR namibia* OR nauruan* OR nepal* OR nicaragua* OR niger OR nigeria* OR nigerien* OR oman* OR muscat OR pakistan* OR panama* OR paraguay* OR peru OR peruvian* OR philippin* OR philipin* OR phillipin* OR phillippin* OR filipin* OR poland OR polish OR pole OR poles OR romania* OR russia* OR ussr OR soviet-union* OR union-of-soviet-socialist-republic* OR rwand* OR ruand* OR samoa* OR pacific-island* OR polynesia* OR sao-tome* OR senegal* OR serbia* OR seychell* OR sierra-leone* OR slovak* OR melanesia* OR solomon-island* OR norfolk-island* OR somali* OR sri-lanka* OR ceylon* OR saint-kitt* OR st-kitt* OR kittitian* OR saint-lucia* OR st-lucia* OR saint-vincent* OR st-vincent* OR vincentian* OR grenadin* OR sudan* OR surinam* OR syria* OR tadjik* OR tadzhik* OR tajik* OR tanzania* OR tanganyika* OR thailand* OR thai OR thais OR siam OR timor* OR togo OR togolese* OR tonga* OR trinidad* OR tobago* OR tunisia* OR turkey OR turk OR turks OR turkmen* OR uganda* OR ukrain* OR uruguay* OR uzbek* OR vanuatu* OR new-hebride* OR venezuela* OR vietnam* OR viet-nam OR viet-names* OR middle-east* OR west-bank OR gaza OR palestin* OR yemen* OR yugoslav* OR zambia* OR zimbabwe* OR rhodesia* OR global-south OR magreb* OR maghrib* OR sahara* OR west-indies OR caribbean* OR central-america* OR latin-america* OR south-america* OR central-asia* OR north-asia* OR northern-asia* OR southeastern-asia* OR south-eastern-asia* OR southeast-asia* OR south-east-asia* OR west-asia* OR western-asia* OR east-europe* OR eastern-europe* OR lmic OR lmics OR third-world OR lami-countr* OR transitional-countr* OR ((developing OR less-developed OR lesser-developed OR under-developed OR underdeveloped OR middle-income OR low-income OR lower-income OR underserved OR under-served OR deprived OR poor OR poorer) W/3 (countr* OR nation* OR population* OR world* OR econom*)) OR ((low OR lower) W/3 (gdp OR gnp OR gross-domestic OR gross-national)) OR ((emerging) W/3 (econom* OR nation*)))) OR (AFFIL(afghan* OR africa* OR albania* OR algeria* OR angola* OR argentin* OR armenia* OR azerbaijan* OR bangladesh* OR bangalee* OR bajan* OR belarus* OR byelarus* OR belorus* OR byelorus* OR belize* OR benin* OR dahomey OR bhutan* OR bolivia* OR bosnia* OR herzegovin* OR botswana* OR batswana* OR bechuanaland* OR brazil* OR brasil* OR bulgaria* OR burkina* OR upper-volta OR burundi* OR urundi* OR cabo-verde* OR cape-verde* OR cambodia* OR kampuchea* OR khmer* OR cameroon* OR cameron* OR cameroun* OR ubangi* OR chad OR chadian* OR chile OR chilean* OR china OR chinese OR colombia* OR comoro* OR comorian* OR mayotte* OR congo OR congolese OR zaire* OR costa-rica* OR cote-d-ivoir* OR ivory-coast* OR ivorian* OR croatia* OR cuba* OR djibouti* OR dominica* OR ecuador* OR egypt* OR united-arab-republic* OR salvador* OR equatoguinean* OR eritrea* OR estonia* OR eswatin* OR swazi* OR swati* OR ethiopia* OR fiji* OR gabon* OR gambia* OR georgia* OR ghana* OR gold-coast* OR gibraltar* OR grenad* OR guam* OR guatemala* OR guinea* OR guyan* OR guiana* OR haiti* OR hispaniola OR hondura* OR hungar* OR india* OR indonesia* OR timor* OR iran* OR iraq* OR jamaica* OR jordan* OR kazakh* OR kenya* OR north-korea* OR kosov* OR kyrgyz* OR kirghiz* OR kirgiz* OR kirghiz* OR lao OR laos OR loatian* OR latvia* OR lebanon OR lebanese OR lesoth* OR basutoland OR mosotho* OR basotho* OR liberia* OR libya* OR lithuania* OR macedonia* OR madagasca* OR malagasy* OR malawi* OR nyasaland OR malaysia* OR maldiv* OR mali OR malian OR malians OR micronesia* OR kiribati* OR marshall-island* OR marshallese OR nauru* OR mariana* OR palau* OR tuvalu* OR mauritania* OR mauritian* OR mauritius OR mexico OR mexican* OR moldov* OR mongol* OR montenegr* OR morocc* OR ifni OR mozambi* OR myanma* OR burma* OR burmese* OR namibia* OR nauruan* OR nepal* OR nicaragua* OR niger OR nigeria* OR nigerien* OR oman* OR muscat OR pakistan* OR panama* OR paraguay* OR peru OR peruvian* OR philippin* OR philipin* OR phillipin* OR phillippin* OR filipin* OR poland OR polish OR pole OR poles OR romania* OR russia* OR ussr OR soviet-union* OR union-of-soviet-socialist-republic* OR rwand* OR ruand* OR samoa* OR pacific-island* OR polynesia* OR sao-tome* OR senegal* OR serbia* OR seychell* OR sierra-leone* OR slovak* OR melanesia* OR solomon-island* OR norfolk-island* OR somali* OR sri-lanka* OR ceylon* OR saint-kitt* OR st-kitt* OR kittitian* OR saint-lucia* OR st-lucia* OR saint-vincent* OR st-vincent* OR vincentian* OR grenadin* OR sudan* OR surinam* OR syria* OR tadjik* OR tadzhik* OR tajik* OR tanzania* OR tanganyika* OR thailand* OR thai OR siam OR timor* OR togo OR togolese* OR tonga* OR trinidad* OR tobago* OR tunisia* OR turkey OR turk OR turks OR turkmen* OR uganda* OR ukrain* OR uruguay* OR uzbek* OR vanuatu* OR new-hebride* OR venezuela* OR vietnam* OR viet-nam OR viet-names* OR middle-east* OR west-bank OR gaza OR palestin* OR yemen* OR yugoslav* OR zambia* OR zimbabwe* OR rhodesia* OR global-south OR maghreb* OR maghrib* OR sahara* OR west-indies OR caribbean* OR central-america* OR latin-america* OR south-america* OR central-asia* OR north-asia* OR northern-asia* OR southeastern-asia* OR south-eastern-asia* OR southeast-asia* OR south-east-asia* OR west-asia* OR western-asia* OR east-europe* OR eastern-europe*))) AND (PUBYEAR AFT 2004) AND (INDEX(embase)) AND NOT (KEY((animal* OR nonhuman* OR rat OR rats OR mouse OR mice OR rodent* OR murine* OR primate* OR monkey* OR dog OR dogs OR canine* OR pig* OR porcine*) AND NOT (human* AND (animal* OR nonhuman* OR rat OR rats OR mouse OR mice OR rodent* OR murine* OR primate* OR monkey* OR dog OR dogs OR canine* OR pig* OR porcine*)))) AND NOT (TITLE(editorial* OR comment* OR case-report* OR systematic-review* OR meta-analys* OR ((trial*) AND (protocol*))))

---

**GIM
4910 Results***with publication date limit of 2005-2021*

(tw:(( large-intestine* OR large-bowel* OR cecum* OR coecum* OR caecum* OR colorect* OR colon* OR rectum* OR rectal OR sigmoid* OR abdominoperineal OR splenic-flexur* OR colon-flexur* OR colonic-flexur* OR hepatic-flexur* OR abdominal-perineal* OR abdominoperineal* OR abdomen-perineal*) AND (neoplas* OR cancer* OR adenoma* OR carcinoma* OR adenocarcinoma* OR malignan* OR tumor* OR tumour* OR metasta* OR lesion* OR mass*))) AND (tw:(colectom* OR hemicolectom* OR proctectom* OR proctocolectom* OR coloproctectom* OR surger* OR surgical* OR resection* OR debulk* OR excision*)) AND (tw:(surviv* OR prognos* OR mortalit* OR death* OR dead OR died OR fatal* OR outcome* OR OS OR DFS OR PFS OR MOS)) AND NOT (mh:(Animals AND NOT (Humans AND Animals))) AND NOT (mh:(Editorial OR Comment OR clinical-trial-protocol)) AND NOT (ti:(case-report*OR systematic-review* OR meta-analys*))

---

**Updated Searches**

**PubMed
1,156 Results**((“Intestine, Large”[mesh:noexp] OR “Colon”[mesh] OR “Cecum”[mesh:noexp] OR “Rectum”[mesh] OR (large-intestine*[tiab] OR large-bowel*[tiab] OR cecum*[tiab] OR caecum*[tiab] OR coecum*[tiab] OR colorect*[tiab] OR colon*[tiab] OR rectum*[tiab] OR rectal[tiab] OR sigmoid*[tiab] OR abdominoperineal*[tiab] OR ((flexur*[tiab]) AND (colon*[tiab] OR spleen*[tiab] OR splenic[tiab] OR hepatic[tiab])) OR ((abdomen*[tiab] OR abdomin*[tiab]) AND (perineal*[tiab])))) AND ("Neoplasms"[mesh] OR (neoplas*[tiab] OR cancer*[tiab] OR adenocancer*[tiab] OR adenocarcin*[tiab] OR adenoma*[tiab] OR carcino*[tiab] OR malignan*[tiab] OR tumor*[tiab] OR tumour*[tiab] OR metasta*[tiab] OR cyst*[tiab] OR growth*[tiab] OR lesion*[tiab]))) AND ("Colectomy"[Mesh] OR “Proctectomy”[mesh] OR (surger*[tiab] OR surgery[sh] OR surgical*[tiab] OR resection*[tiab] OR debulk*[tiab] OR excision*[tiab] OR colectom*[tiab] OR hemicolectom*[tiab] OR proctectom*[tiab] OR proctocolectom*[tiab] OR coloproctectom*[tiab])) AND ("Survivorship"[mesh] OR "Survivors"[mesh] OR "Prognosis"[mesh] OR "Survival Analysis"[mesh] OR "Mortality"[mesh] OR "Survival Rate"[mesh] OR (surviv*[tiab] OR prognos*[tiab] OR mortalit*[tiab] OR mortality[sh] OR death*[tiab] OR dead[tiab] OR died[tiab] OR fatal*[tiab] OR outcome*[tiab] OR OS[tiab] OR DFS[tiab] OR PFS[tiab] OR MOS[tiab])) AND ("afghanistan"[mesh] OR "albania"[mesh] OR "algeria"[mesh] OR "american samoa"[mesh] OR "angola"[mesh] OR "argentina"[mesh] OR "armenia"[mesh] OR "azerbaijan"[mesh] OR "bangladesh"[mesh] OR "republic of belarus"[mesh] OR "belize"[mesh] OR "benin"[mesh] OR "bhutan"[mesh] OR "bolivia"[mesh] OR "bosnia and herzegovina"[mesh] OR "botswana"[mesh] OR "brazil"[mesh] OR "bulgaria"[mesh] OR "burkina faso"[mesh] OR "burundi"[mesh] OR "cabo verde"[mesh] OR "cambodia"[mesh] OR "cameroon"[mesh] OR "central african republic"[mesh] OR "chad"[mesh] OR "chile"[mesh] OR "china"[mesh] OR "colombia"[mesh] OR "comoros"[mesh] OR "democratic republic of the congo"[mesh] OR "congo"[mesh] OR "costa rica"[mesh] OR "cote d’ivoire"[mesh] OR "croatia"[mesh] OR "cuba"[mesh] OR "djibouti"[mesh] OR "dominica"[mesh] OR "dominican republic"[mesh] OR "ecuador"[mesh] OR "egypt"[mesh] OR "el salvador"[mesh] OR "equatorial guinea"[mesh] OR "eritrea"[mesh] OR "estonia"[mesh] OR "Eswatini"[Mesh] OR "ethiopia"[mesh] OR "fiji"[mesh] OR "gabon"[mesh] OR "gambia"[mesh] OR "georgia (republic)"[mesh] OR "ghana"[mesh] OR "gibraltar"[mesh] OR "grenada"[mesh] OR "guam"[mesh] OR "guatemala"[mesh] OR "guinea"[mesh] OR "guinea-bissau"[mesh] OR "guyana"[mesh] OR "haiti"[mesh] OR "honduras"[mesh] OR "hungary"[mesh] OR "india"[mesh] OR "indonesia"[mesh] OR "iran"[mesh] OR "iraq"[mesh] OR "jamaica"[mesh] OR "jordan"[mesh] OR "kazakhstan"[mesh] OR "kenya"[mesh] OR "democratic people’s republic of korea"[mesh] OR "kosovo"[mesh] OR "kyrgyzstan"[mesh] OR "laos"[mesh] OR "latvia"[mesh] OR "lebanon"[mesh] OR "lesotho"[mesh] OR "liberia"[mesh] OR "libya"[mesh] OR "lithuania"[mesh] OR "republic of north macedonia"[mesh] OR "madagascar"[mesh] OR "malawi"[mesh] OR "malaysia"[mesh] OR "indian ocean islands"[mesh] OR "mali"[mesh] OR "micronesia"[mesh] OR "palau"[mesh] OR "mauritania"[mesh] OR "mauritius"[mesh] OR "mexico"[mesh] OR "moldova"[mesh] OR "mongolia"[mesh] OR "montenegro"[mesh] OR "morocco"[mesh] OR "mozambique"[mesh] OR "myanmar"[mesh] OR "namibia"[mesh] OR "nepal"[mesh] OR "nicaragua"[mesh] OR "niger"[mesh] OR "nigeria"[mesh] OR "oman"[mesh] OR "pakistan"[mesh] OR "panama"[mesh] OR "papua new guinea"[mesh] OR "paraguay"[mesh] OR "peru"[mesh] OR "philippines"[mesh] OR "poland"[mesh] OR "romania"[mesh] OR "russia"[mesh] OR "rwanda"[mesh] OR "samoa"[mesh] OR "sao tome and principe"[mesh] OR "senegal"[mesh] OR "serbia"[mesh] OR "seychelles"[mesh] OR "sierra leone"[mesh] OR "slovakia"[mesh] OR "slovenia"[mesh] OR "melanesia"[mesh] OR "somalia"[mesh] OR "south africa"[mesh] OR "south sudan"[mesh] OR "sri lanka"[mesh] OR "saint kitts and nevis"[mesh] OR "saint lucia"[mesh] OR "saint vincent and the grenadines"[mesh] OR "sudan"[mesh] OR "suriname"[mesh] OR "syria"[mesh] OR "tajikistan"[mesh] OR "tanzania"[mesh] OR "thailand"[mesh] OR "timor-leste"[mesh] OR "togo"[mesh] OR "tonga"[mesh] OR "trinidad and tobago"[mesh] OR "tunisia"[mesh] OR "turkey"[mesh] OR "turkmenistan"[mesh] OR "uganda"[mesh] OR "ukraine"[mesh] OR "uruguay"[mesh] OR "uzbekistan"[mesh] OR "vanuatu"[mesh] OR "venezuela"[mesh] OR "vietnam"[mesh] OR "middle east"[mesh] OR "yemen"[mesh] OR "yugoslavia"[mesh] OR "zambia"[mesh] OR "zimbabwe"[mesh] OR "africa south of the sahara"[mesh] OR "africa, central"[mesh] OR "africa, northern"[mesh] OR "africa, southern"[mesh] OR "africa, eastern"[mesh] OR "africa, western"[mesh] OR "west indies"[mesh] OR "indian ocean islands"[mesh] OR "caribbean region"[mesh] OR "central america"[mesh] OR "latin america"[mesh] OR "south america"[mesh] OR "asia, central"[mesh] OR "asia, northern"[mesh] OR "asia, southeastern"[mesh] OR "asia, western"[mesh] OR "europe, eastern"[mesh] OR "developing countries"[mesh] OR (afghan*[tiab] OR africa[tiab] OR african[tiab] OR africans[tiab] OR albania[tiab] OR albanian[tiab] OR albanians[tiab] OR algeria[tiab] OR algerian[tiab] OR algerians[tiab] OR angola[tiab] OR angolan[tiab] OR angolans[tiab] OR argentina[tiab] OR argentine[tiab] OR argentines[tiab] OR argentinian[tiab] OR argentinians[tiab] OR armenia[tiab] OR armenian[tiab] OR armenians[tiab] OR azerbaijan[tiab] OR azerbaijani[tiab] OR azerbaijanis[tiab] OR bangladesh[tiab] OR bangladeshi[tiab] OR bangladeshis[tiab] OR bangalee[tiab] OR bajan[tiab] OR belarus*[tiab] OR byelarus*[tiab] OR belorus*[tiab] OR byelorus*[tiab] OR belize[tiab] OR belizean[tiab] OR benin[tiab] OR beninese[tiab] OR benineses[tiab] OR dahomey[tiab] OR bhutan[tiab] OR bhutanese[tiab] OR bolivia[tiab] OR bolivian[tiab] OR bolivians[tiab] OR bosnia[tiab] OR bosnian[tiab] OR bosnians[tiab] OR herzegovina[tiab] OR herzegovinian[tiab] OR herzegovinians[tiab] OR botswana[tiab] OR botswanan[tiab] OR botswanans[tiab] OR batswana[tiab] OR bechuanaland[tiab] OR brazil[tiab] OR brazilian[tiab] OR brazilians[tiab] OR brasil[tiab] OR brasilian[tiab] OR brasilians[tiab] OR bulgaria[tiab] OR bulgarian[tiab] OR bulgarians[tiab] OR burkina[tiab] OR burkinabe[tiab] OR burkinabes[tiab] OR upper-volta[tiab] OR burundi[tiab] OR burundian[tiab] OR urundi[tiab] OR cabo-verde[tiab] OR cape-verde[tiab] OR cape-verdean[tiab] OR cape-verdeans[tiab] OR cambodia[tiab] OR cambodian[tiab] OR cambodians[tiab] OR kampuchea[tiab] OR kampuchean[tiab] OR kampucheans[tiab] OR khmer[tiab] OR khmers[tiab] OR cameroon[tiab] OR cameroons[tiab] OR cameroonian[tiab] OR cameroonians[tiab] OR cameroonese[tiab] OR cameron[tiab] OR cameronian[tiab] OR cameronians[tiab] OR cameroun[tiab] OR ubangi[tiab] OR chad[tiab] OR chadian[tiab] OR chadians[tiab] OR chile[tiab] OR chilean[tiab] OR chileans[tiab] OR china[tiab] OR chinese[tiab] OR colombia[tiab] OR colombian[tiab] OR colombians[tiab] OR colombiano[tiab] OR colombianos[tiab] OR comoro[tiab] OR comoros[tiab] OR mayotte[tiab] OR congo[tiab] OR congolese[tiab] OR zaire[tiab] OR zairean[tiab] OR zaireans[tiab] OR zairese[tiab] OR costa-rica[tiab] OR costa-rican[tiab] OR costa-ricans[tiab] OR cote-d-ivoire[tiab] OR ivory-coast[tiab] OR ivorian[tiab] OR ivorians[tiab] OR croatia[tiab] OR croatian[tiab] OR croatians[tiab] OR cuba[tiab] OR cuban[tiab] OR cubans[tiab] OR djibouti[tiab] OR djiboutian[tiab] OR djiboutians[tiab] OR dominica[tiab] OR dominican[tiab] OR dominicans[tiab] OR ecuador[tiab] OR ecuadorian[tiab] OR ecuadorians[tiab] OR egypt[tiab] OR egyptian[tiab] OR egyptians[tiab] OR united-arab-republic[tiab] OR salvador[tiab] OR salvadoran[tiab] OR salvadorans[tiab] OR equatoguinean[tiab] OR equatoguineans[tiab] OR eritrea[tiab] OR eritrean[tiab] OR eritreans[tiab] OR estonia[tiab] OR estonian[tiab] OR estonians[tiab] OR eswatini[tiab] OR swaziland[tiab] OR swazi[tiab] OR swazis[tiab] OR swati[tiab] OR ethiopia[tiab] OR ethiopian[tiab] OR ethiopians[tiab] OR fiji[tiab] OR fijian[tiab] OR fijians[tiab] OR gabon[tiab] OR gabonese[tiab] OR gambia[tiab] OR gambian[tiab] OR gambians[tiab] OR georgia[tiab] OR georgian[tiab] OR georgians[tiab] OR ghana[tiab] OR ghanaian[tiab] OR ghanaians[tiab] OR gold-coast[tiab] OR gibraltar[tiab] OR gibraltarian[tiab] OR gibraltarians[tiab] OR grenada[tiab] OR grenadian[tiab] OR grenadians[tiab] OR guam[tiab] OR guamanian[tiab] OR guamanians[tiab] OR guatemala[tiab] OR guatemalan[tiab] OR guatemalans[tiab] OR guinea[tiab] OR guinean[tiab] OR guineans[tiab] OR guyana[tiab] OR guyanese[tiab] OR guiana[tiab] OR guianan[tiab] OR haiti[tiab] OR haitian[tiab] OR haitians[tiab] OR hispaniola[tiab] OR honduras[tiab] OR honduran[tiab] OR hondurans[tiab] OR hungary[tiab] OR hungarian[tiab] OR hungarians[tiab] OR india[tiab] OR indian[tiab] OR indians[tiab] OR indonesia[tiab] OR indonesia[tiab] OR indonesia[tiab] OR iran[tiab] OR irani[tiab] OR iranis[tiab] OR iranian[tiab] OR iranians[tiab] OR iraq[tiab] OR iraqi[tiab] OR iraqis[tiab] OR iraqian[tiab] OR jamaica[tiab] OR jamaican[tiab] OR jamaicans[tiab] OR jordan[tiab] OR jordanian[tiab] OR jordanians[tiab] OR kazak*[tiab] OR kenya[tiab] OR kenyan[tiab] OR kenyans[tiab] OR north-korea[tiab] OR north-korean[tiab] OR north-koreans[tiab] OR democratic-people-s-republic-of-korea[tiab] OR kosov*[tiab] OR kirgiz*[tiab] OR kyrgyz*[tiab] OR kirghiz*[tiab] OR lao[tiab] OR laos[tiab] OR loatian*[tiab] OR latvia[tiab] OR latvian[tiab] OR latvians[tiab] OR lebanon[tiab] OR lebanese[tiab] OR lesotho[tiab] OR basutoland[tiab] OR mosotho[tiab] OR basotho[tiab] OR liberia[tiab] OR liberian[tiab] OR liberians[tiab] OR libya[tiab] OR libyan[tiab] OR libyans[tiab] OR lithuania[tiab] OR lithuanian[tiab] OR lithuanians[tiab] OR macedonia[tiab] OR macedonian[tiab] OR macedonians[tiab] OR madagascar[tiab] OR madagascan[tiab] OR madagascans[tiab] OR malagasy[tiab] OR malawi[tiab] OR malawian[tiab] OR malawians[tiab] OR nyasaland[tiab] OR malaysia*[tiab] OR maldiv*[tiab] OR mali[tiab] OR malian*[tiab] OR malians[tiab] OR micronesia*[tiab] OR kiribati[tiab] OR marshall-island*[tiab] OR marshallese[tiab] OR mariana[tiab] OR marianas[tiab] OR palau[tiab] OR palauan[tiab] OR palauans[tiab] OR tuvalu[tiab] OR tuvaluan[tiab] OR tuvaluans[tiab] OR mauritania[tiab] OR mauritanian[tiab] OR mauritanians[tiab] OR mauritius[tiab] OR mauritian[tiab] OR mauritians[tiab] OR mexico[tiab] OR mexican[tiab] OR mexicans[tiab] OR moldova[tiab] OR moldovan[tiab] OR moldovans[tiab] OR mongolia[tiab] OR mongolian[tiab] OR mongolians[tiab] OR mongol[tiab] OR mongols[tiab] OR montenegro[tiab] OR montenegrin[tiab] OR montenegrins[tiab] OR morocco[tiab] OR moroccan[tiab] OR moroccans[tiab] OR mozambique[tiab] OR mozambican[tiab] OR mozambicans[tiab] OR myanmar[tiab] OR myanma[tiab] OR myanmarese[tiab] OR burma[tiab] OR burmese[tiab] OR namibia[tiab] OR namibian[tiab] OR namibians[tiab] OR nauru[tiab] OR nauruan[tiab] OR nauruans[tiab] OR nepal[tiab] OR nepali[tiab] OR nepalis[tiab] OR nepalese[tiab] OR nicaragua[tiab] OR nicaraguan[tiab] OR nicaraguans[tiab] OR niger[tiab] OR nigeria[tiab] OR nigerian[tiab] OR nigerians[tiab] OR nigerien[tiab] OR nigeriens[tiab] OR oman[tiab] OR omani[tiab] OR omanis[tiab] OR muscat[tiab] OR pakistan[tiab] OR pakistani[tiab] OR pakistanis[tiab] OR panama[tiab] OR panamanian[tiab] OR panamanians[tiab] OR paraguay[tiab] OR paraguayan[tiab] OR paraguayans[tiab] OR peru[tiab] OR peruvian[tiab] OR peruvians[tiab] OR philippin*[tiab] OR philipin*[tiab] OR phillipin*[tiab] OR phillippin*[tiab] OR filipin*[tiab] OR poland[tiab] OR polish[tiab] OR pole[tiab] OR poles[tiab] OR romania[tiab] OR romanian[tiab] OR romanians[tiab] OR russia[tiab] OR russian[tiab] OR russians[tiab] OR ussr[tiab] OR soviet[tiab] OR soviets[tiab] OR rwand*[tiab] OR ruand*[tiab] OR samoa[tiab] OR samoan[tiab] OR samoans[tiab] OR pacific-island[tiab] OR pacific-islands[tiab] OR pacific-islander[tiab] OR pacific-islanders[tiab] OR polynesia[tiab] OR polynesian[tiab] OR polynesians[tiab] OR sao-tome*[tiab] OR senegal[tiab] OR senegalese[tiab] OR serbia[tiab] OR serbian[tiab] OR serbians[tiab] OR seychelles[tiab] OR seychellois[tiab] OR seychelloise[tiab] OR sierra-leone[tiab] OR sierra-leonean[tiab] OR sierra-leoneans[tiab] OR slovak[tiab] OR slovaks[tiab] OR slovakia[tiab] OR slovakian[tiab] OR slovakians[tiab] OR slovenia[tiab] OR slovenian[tiab] OR slovenians[tiab] OR slovene[tiab] OR slovenes[tiab] OR melanesia[tiab] OR melanesian[tiab] OR melanesians[tiab] OR solomon-island[tiab] OR solomon-islands[tiab] OR solomon-islander[tiab] OR solomon-islanders[tiab] OR norfolk-island*[tiab] OR somali[tiab] OR somalian[tiab] OR somalians[tiab] OR sri-lanka[tiab] OR sri-lankan[tiab] OR sri-lankans[tiab] OR ceylon[tiab] OR ceylonese[tiab] OR saint-kitts[tiab] OR st-kitts[tiab] OR kittitian[tiab] OR kittitians[tiab] OR saint-lucia[tiab] OR saint-lucian[tiab] OR st-lucia[tiab] OR saint-vincent[tiab] OR st-vincent[tiab] OR vincentian[tiab] OR vincentians[tiab] OR grenadines[tiab] OR sudan[tiab] OR sudanese[tiab] OR surinam[tiab] OR suriname[tiab] OR surinamese[tiab] OR syria[tiab] OR syrian[tiab] OR syrians[tiab] OR tadjikistan[tiab] OR tadzhikistan[tiab] OR tadzhik[tiab] OR tajikistan[tiab] OR tajik[tiab] OR tajikistani[tiab] OR tanzania[tiab] OR tanzanian[tiab] OR tanzanians[tiab] OR tanganyikan[tiab] OR thailand[tiab] OR thai[tiab] OR thais[tiab] OR siam[tiab] OR timor[tiab] OR timorese[tiab] OR timoreses[tiab] OR togo[tiab] OR togolese[tiab] OR tonga[tiab] OR tongan[tiab] OR tongans[tiab] OR trinidad[tiab] OR trinidadian[tiab] OR trinidadians[tiab] OR tobagonian[tiab] OR tobagonians[tiab] OR tunisia[tiab] OR tunisian[tiab] OR tunisians[tiab] OR turkey[tiab] OR turk[tiab] OR turks[tiab] OR turkish[tiab] OR turkmenistan[tiab] OR turkmen[tiab] OR turkmens[tiab] OR uganda[tiab] OR ugandan[tiab] OR ugandans[tiab] OR ukraine[tiab] OR ukrainian[tiab] OR ukrainians[tiab] OR uruguay[tiab] OR uruguayan[tiab] OR uruguayans[tiab] OR uzbekistan[tiab] OR uzbek[tiab] OR uzbeks[tiab] OR vanuatu[tiab] vanuatuan[tiab] OR vanuatuans[tiab] OR new-hebrides[tiab] OR venezuela[tiab] OR venezuelan[tiab] OR venezuelans[tiab] OR vietnam[tiab] OR vietnamese[tiab] OR viet-nam[tiab] OR viet-namese[tiab] OR middle-east[tiab] OR middle-eastern[tiab] OR west-bank[tiab] OR gaza[tiab] OR palestine[tiab] OR palestinian[tiab] OR palestinians[tiab] OR yemen*[tiab] OR yugoslav*[tiab] OR zambia[tiab] OR zambian[tiab] OR zambians[tiab] OR zimbabwe[tiab] OR zimbabwean[tiab] OR zimbabweans[tiab] OR rhodesia[tiab] OR rhodesian[tiab] OR rhodesians[tiab] OR global-south[tiab] OR maghreb[tiab] OR maghrib[tiab] OR sahara*[tiab] OR west-indies[tiab] OR caribbean[tiab] OR caribbeans[tiab] OR central-america[tiab] OR central-american[tiab] OR central-americans[tiab] OR latin-america[tiab] OR latin-american[tiab] OR latin-americans[tiab] OR south-america[tiab] OR south-american[tiab] OR south-americans[tiab] OR central-asia[tiab] OR central-asian[tiab] OR central-asians[tiab] OR north-asia[tiab] OR north-asian[tiab] OR north-asians[tiab] OR northern-asia[tiab] OR northern-asian[tiab] OR northern-asians[tiab] OR southeastern-asia[tiab] OR southeastern-asian[tiab] OR southeastern-asians[tiab] OR south-eastern-asia[tiab] OR south-eastern-asian[tiab] OR southeast-asia[tiab] OR southeast-asian[tiab]OR southeast-asians[tiab]OR south-east-asia[tiab] OR south-east-asian[tiab] OR south-east-asians[tiab] OR west-asia[tiab] OR west-asian[tiab] OR west-asians[tiab] OR western-asia[tiab] OR western-asian[tiab] OR western-asians[tiab] OR east-europe[tiab] OR east-european[tiab] OR east-europeans[tiab] OR eastern-europe[tiab] OR eastern-european[tiab] OR eastern-europeans[tiab] OR afghan*[ad] OR africa[ad] OR african[ad] OR albania[ad] OR albanian[ad] OR algeria[ad] OR algerian[ad] OR angola[ad] OR angolan[ad] OR argentina[ad] OR argentine[ad] OR argentinian[ad] OR armenia[ad] OR armenian[ad] OR azerbaijan[ad] OR azerbaijani[ad] OR bangladesh[ad] OR bangladeshi[ad] OR belarus*[ad] OR byelarus*[ad] OR belorus*[ad] OR byelorus*[ad] OR belize[ad] OR benin[ad] OR beninese[ad] OR dahomey[ad] OR bhutan[ad] OR bhutanese[ad] OR bolivia[ad] OR bolivian[ad] OR bosnia[ad] OR bosnian[ad] OR herzegovina[ad] OR herzegovinian[ad] OR botswana[ad] OR brazil[ad] OR brazilian[ad] OR brasil[ad] OR brasilian[ad] OR bulgaria[ad] OR bulgarian[ad] OR burkina[ad] OR burkinabe[ad] OR burundi[ad] OR cabo-verde[ad] OR cape-verde[ad] OR cambodia[ad] OR cambodian[ad] OR kampuchea[ad] OR khmer[ad] OR cameroon[ad] OR cameron[ad] OR cameroun[ad] OR chad[ad] OR chadian[ad] OR chile[ad] OR chilean[ad] OR china[ad] OR chinese[ad] OR colombia[ad] OR colombian[ad] OR comoro[ad] OR comoros[ad] OR mayotte[ad] OR congo[ad] OR congolese[ad] OR zaire[ad] OR zairean[ad] OR costa-rica[ad] OR costa-rican[ad] OR cote-d-ivoire[ad] OR ivory-coast[ad] OR ivorian[ad] OR croatia[ad] OR croatian[ad] OR cuba[ad] OR cuban[ad] OR djibouti[ad] OR djiboutian[ad] OR dominica[ad] OR dominican[ad] OR ecuador[ad] OR ecuadorian[ad] OR egypt[ad] OR egyptian[ad] OR united-arab-republic[ad] OR salvador[ad] OR salvadoran[ad] OR eritrea[ad] OR eritrean[ad] OR estonia[ad] OR eswatini[ad] OR swaziland[ad] OR ethiopia[ad] OR ethiopian[ad] OR fiji[ad] OR fijian[ad] OR gabon[ad] OR gambia[ad] OR gambian[ad] OR georgia[ad] OR georgian[ad] OR ghana[ad] OR ghanaian[ad] OR gibraltar[ad] OR grenada[ad] OR guam[ad] OR guatemala[ad] OR guatemalan[ad] OR guinea[ad] OR guinean[ad] OR guyana[ad] OR guiana[ad] OR guianan[ad] OR haiti[ad] OR haitian[ad] OR hispaniola[ad] OR honduras[ad] OR honduran[ad] OR hungary[ad] OR hungarian[ad] OR india[ad] OR indian[ad] OR indians[ad] OR indonesia[ad] OR indonesian[ad] OR iran[ad] OR iranian[ad] OR iraq[ad] OR iraqi[ad] OR jamaica[ad] OR jamaican[ad] OR jordan[ad] OR jordanian[ad] OR kazakhstan[ad] OR kazakstan[ad] OR kenya[ad] OR kenyan[ad] OR north-korea[ad] OR north-korean[ad] OR kosov*[ad] OR kirgiz*[ad] OR kyrgyz*[ad] OR kirghiz*[ad] OR lao[ad] OR laos[ad] OR latvia[ad] OR latvian[ad] OR lebanon[ad] OR lebanese[ad] OR lesotho[ad] OR basotho[ad] OR liberia[ad] OR liberian[ad] OR libya[ad] OR libyan[ad] OR lithuania[ad] OR lithuanian[ad] OR macedonia[ad] OR macedonian[ad] OR madagascar[ad] OR malagasy[ad] OR malawi[ad] OR malawian[ad] OR malaysia*[ad] OR maldiv*[ad] OR mali[ad] OR malian[ad] OR malians[ad] OR micronesia*[ad] OR kiribati[ad] OR marshall-islands[ad] OR marshallese[ad] OR nauru[ad] OR mariana[ad] OR marianas[ad] OR palau[ad] OR tuvalu[ad] OR mauritania[ad] OR mauritanian[ad] OR mauritius[ad] OR mauritian[ad] OR mexico[ad] OR mexican[ad] OR moldova[ad] OR moldovan[ad] OR mongolia[ad] OR mongolian[ad] OR mongol[ad] OR montenegro[ad] OR montenegrin[ad] OR morocco[ad] OR moroccan[ad] OR mozambique[ad] OR mozambican[ad] OR myanmar[ad] OR myanma[ad] OR burma[ad] OR burmese[ad] OR burmese[ad] OR namibia[ad] OR namibian[ad] OR nepal[ad] OR nepalese[ad] OR nicaragua[ad] OR nicaraguan[ad] OR niger[ad] OR nigeria[ad] OR nigerian[ad] OR oman[ad] OR omani[ad] OR muscat[ad] OR pakistan[ad] OR pakistani[ad] OR panama[ad] OR panamanian[ad] OR paraguay[ad] OR paraguayan[ad] OR peru[ad] OR peruvian*[ad] OR philippin*[ad] OR philipin*[ad] OR phillipin*[ad] OR phillippin*[ad] OR filipin*[ad] OR poland[ad] OR polish[ad] OR rhodesia[ad] OR rhodesian[ad] OR romania[ad] OR romanian[ad] OR russia[ad] OR russian[ad] OR ussr[ad] OR soviet[ad] OR rwand*[ad] OR ruand*[ad] OR samoa[ad] OR samoan[ad] OR pacific-island[ad] OR pacific-islands[ad] OR pacific-islander[ad] OR pacific-islanders[ad] OR polynesia[ad] OR polynesian[ad] OR sao-tome-and-principe[ad] OR sao-tome[ad] OR senegal[ad] OR senegalese[ad] OR serbia[ad] OR serbian[ad] OR seychelles[ad] OR sierra-leone[ad] OR slovak[ad] OR slovakia[ad] OR slovakian[ad] OR slovenia[ad] OR slovenian[ad] OR slovene[ad] OR melanesia[ad] OR melanesian[ad] OR solomon-island[ad] OR solomon-islands[ad] OR norfolk-island[ad] OR somalia[ad] OR sri-lanka[ad] OR sri-lankan[ad] OR ceylon[ad] OR saint-kitts[ad] OR st-kitts[ad] OR saint-lucia[ad] OR st-lucia[ad] OR saint-vincent[ad] OR st-vincent[ad] OR grenadines[ad] OR sudan[ad] OR sudanese[ad] OR surinam[ad] OR suriname[ad] OR syria[ad] OR syrian[ad] OR tadjikistan[ad] OR tadzhikistan[ad] OR tadzhik[ad] OR tajikistan[ad] OR tajik[ad] OR tanzania[ad] OR tanzanian[ad] OR thailand[ad] OR thai[ad] OR siam[ad] OR timor[ad] OR togo[ad] OR togolese[ad] OR tonga[ad] OR tongan[ad] OR trinidad[ad] OR tobago[ad] OR tunisia[ad] OR tunisian[ad] OR turkey[ad] OR turk[ad] OR turks[ad] OR turkish[ad] OR turkmenistan[ad] OR turkmen[ad] OR uganda[ad] OR ugandan[ad] OR ukraine[ad] OR ukrainian[ad] OR uruguay[ad] OR uruguayan[ad] OR uzbekistan[ad] OR uzbek[ad] OR vanuatu[ad] OR venezuela[ad] OR venezuelan[ad] OR vietnam[ad] OR vietnamese[ad] OR viet-nam[ad] OR viet-namese[ad] OR middle-east[ad] OR middle-eastern[ad] OR west-bank[ad] OR gaza[ad] OR palestine[ad] OR palestinian[ad] OR yemen[ad] OR yemeni[ad] OR yugoslav*[ad] OR zambia[ad] OR zambian[ad] OR zimbabwe[ad] OR global-south[ad] OR sahara*[ad] OR west-indies[ad] OR caribbean[ad] OR central-america*[ad] OR central-american[ad] OR latin-america[ad] OR latin-american[ad] OR south-america[ad] OR south-american[ad] OR central-asia[ad] OR central-asian[ad] OR north-asia[ad] OR northern-asia[ad] OR southeastern-asia[ad] OR southeast-asia[ad] OR southeast-asian[ad] OR south-east-asia[ad] OR south-east-asian[ad] OR west-asia[ad] OR west-asian[ad] OR western-asia[ad] OR east-europe[ad] OR east-european[ad] OR eastern-europe[ad] OR eastern-european[ad] OR lmic[tiab] OR lmics[tiab] OR third-world[tiab] OR lami-countr*[tiab] OR transitional-countr*[tiab] OR ((developing[tiab] OR less-developed[tiab] OR lesser-developed[tiab] OR under-developed[tiab] OR underdeveloped[tiab] OR middle-income[tiab] OR low-income[tiab] OR lower-income[tiab] OR underserved[tiab] OR under-served[tiab] OR deprived[tiab] OR poor[tiab] OR poorer[tiab] OR improverished[tiab]) AND (country[tiab] OR countries[tiab] OR nation[tiab] OR nations[tiab] OR population[tiab] OR populations[tiab] OR world*[tiab] OR economy[tiab] OR economies[tiab])) OR ((low[tiab] OR lower[tiab]) AND (gdp[tiab] OR gnp[tiab] OR gross-domestic[tiab] OR gross-national[tiab])) OR ((emerging[tiab]) AND (econom*[tiab] OR nation*[tiab])))) NOT ("Animals"[Mesh] NOT ("Animals"[Mesh] AND "Humans"[Mesh])) NOT ("Editorial"[pt] OR "Comment"[pt] OR “Clinical Trial Protocol”[pt] OR case-report*[ti] OR systematic-review*[ti] OR meta-analys*[ti]) AND (2005/06/01:3000[pdat])

---

**Web of Science
723 Results**(TS=(((large-intestine* OR large-bowel* OR cecum* OR c?ecum* OR colorect* OR colon* OR rectum* OR rectal OR sigmoid* OR abdominoperineal*) NEAR/3 (neoplas* OR cancer* OR adenoma* OR carcinoma* OR adenocancer* OR adenocarcinoma* OR malignan* OR tumor* OR tumour* OR metasta* OR lesion* OR mass*) )) OR TS=(((perineal*) AND (abdomen OR abdomin*) ) AND (neoplas* OR cancer* OR adenoma* OR carcinoma* OR adenocancer* OR adenocarcinoma* OR malignan* OR tumor* OR tumour* OR metasta* OR lesion* OR mass*) ) OR TS=(((flexur*) AND (colon* OR spleen* OR splenic OR hepatic*) ) AND (neoplas* OR cancer* OR adenoma* OR carcinoma* OR adenocancer* OR adenocarcinoma* OR malignan* OR tumor* OR tumour* OR metasta* OR lesion* OR mass*))) AND (TS=(colectom* OR hemicolectom* OR proctectom* OR proctocolectom* OR coloproctectom* OR surger* OR surgical* OR resection* OR debulk* OR excision*)) AND (TS=(surviv* OR prognos* OR mortalit* OR death* OR dead OR died OR fatal* OR outcome* OR OS OR DFS OR PFS OR MOS)) AND ((TS=(afghan* OR africa* OR albania* OR algeria* OR angola* OR argentin* OR armenia* OR azerbaijan* OR bangladesh* OR bangalee* OR bajan* OR belarus* OR byelarus* OR belorus* OR byelorus* OR belize* OR benin* OR dahomey OR bhutan* OR bolivia* OR bosnia* OR herzegovin* OR botswana* OR batswana* OR bechuanaland* OR brazil* OR brasil* OR bulgaria* OR burkina* OR upper-volta OR burundi* OR urundi* OR cabo-verde* OR cape-verde* OR cambodia* OR kampuchea* OR khmer* OR cameroon* OR cameron* OR cameroun* OR ubangi* OR chad OR chadian* OR chile OR chilean* OR china OR chinese OR colombia* OR comoro* OR comorian* OR mayotte* OR congo OR congolese OR zaire* OR costa-rica* OR cote-d-ivoir* OR ivory-coast* OR ivorian* OR croatia* OR cuba* OR djibouti* OR dominica* OR ecuador* OR egypt* OR united-arab-republic* OR salvador* OR equatoguinean* OR eritrea* OR estonia* OR eswatin* OR swazi* OR swati* OR ethiopia* OR fiji* OR gabon* OR gambia* OR georgia* OR ghana* OR gold-coast* OR gibraltar* OR grenad* OR guam* OR guatemala* OR guinea* OR guyan* OR guiana* OR haiti* OR hispaniola OR hondura* OR hungar* OR india* OR indonesia* OR timor* OR iran* OR iraq* OR jamaica* OR jordan* OR kazakh* OR kenya* OR north-korea* OR kosov* OR kyrgyz* OR kirghiz* OR kirgiz* OR kirghiz* OR lao OR laos OR loatian* OR latvia* OR lebanon OR lebanese OR lesoth* OR basutoland OR mosotho* OR basotho* OR liberia* OR libya* OR lithuania* OR macedonia* OR madagasca* OR malagasy* OR malawi* OR nyasaland OR malaysia* OR maldiv* OR mali OR malian OR malians OR micronesia* OR kiribati* OR marshall-island* OR marshallese OR nauru* OR mariana* OR palau* OR tuvalu* OR mauritania* OR mauritian* OR mauritius OR mexico OR mexican* OR moldov* OR mongol* OR montenegr* OR morocc* OR ifni OR mozambi* OR myanma* OR burma* OR burmese* OR namibia* OR nauruan* OR nepal* OR nicaragua* OR niger OR nigeria* OR nigerien* OR oman* OR muscat OR pakistan* OR panama* OR paraguay* OR peru OR peruvian* OR philippin* OR philipin* OR phillipin* OR phillippin* OR filipin* OR poland OR polish OR pole OR poles OR romania* OR russia* OR ussr OR soviet-union* OR union-of-soviet-socialist-republic* OR rwand* OR ruand* OR samoa* OR pacific-island* OR polynesia* OR sao-tome* OR senegal* OR serbia* OR seychell* OR sierra-leone* OR slovak* OR melanesia* OR solomon-island* OR norfolk-island* OR somali* OR sri-lanka* OR ceylon* OR saint-kitt* OR st-kitt* OR kittitian* OR saint-lucia* OR st-lucia* OR saint-vincent* OR st-vincent* OR vincentian* OR grenadin* OR sudan* OR surinam* OR syria* OR tadjik* OR tadzhik* OR tajik* OR tanzania* OR tanganyika* OR thailand* OR thai OR thais OR siam OR timor* OR togo OR togolese* OR tonga* OR trinidad* OR tobago* OR tunisia* OR turkey OR turk OR turks OR turkmen* OR uganda* OR ukrain* OR uruguay* OR uzbek* OR vanuatu* OR new-hebride* OR venezuela* OR vietnam* OR viet-nam OR viet-names* OR middle-east* OR west-bank OR gaza OR palestin* OR yemen* OR yugoslav* OR zambia* OR zimbabwe* OR rhodesia* OR global-south OR maghreb* OR maghrib* OR sahara* OR west-indies OR caribbean* OR central-america* OR latin-america* OR south-america* OR central-asia* OR north-asia* OR northern-asia* OR southeastern-asia* OR south-eastern-asia* OR southeast-asia* OR south-east-asia* OR west-asia* OR western-asia* OR east-europe* OR eastern-europe* OR lmic OR lmics OR third-world OR lami-countr* OR transitional-countr* OR ((developing OR less-developed OR lesser-developed OR under-developed OR underdeveloped OR middle-income OR low-income OR lower-income OR underserved OR under-served OR deprived OR poor OR poorer) NEAR/3 (countr* OR nation* OR population* OR world* OR econom*)) OR ((low OR lower) NEAR/3 (gdp OR gnp OR gross-domestic OR gross-national)) OR ((emerging) NEAR/3 (econom* OR nation*)))) OR (CU=(afghan* OR africa* OR albania* OR algeria* OR angola* OR argentin* OR armenia* OR azerbaijan* OR bangladesh* OR bangalee* OR bajan* OR belarus* OR byelarus* OR belorus* OR byelorus* OR belize* OR benin* OR dahomey OR bhutan* OR bolivia* OR bosnia* OR herzegovin* OR botswana* OR batswana* OR bechuanaland* OR brazil* OR brasil* OR bulgaria* OR burkina* OR upper-volta OR burundi* OR urundi* OR cabo-verde* OR cape-verde* OR cambodia* OR kampuchea* OR khmer* OR cameroon* OR cameron* OR cameroun* OR ubangi* OR chad OR chadian* OR chile OR chilean* OR china OR chinese OR colombia* OR comoro* OR comorian* OR mayotte* OR congo OR congolese OR zaire* OR costa-rica* OR cote-d-ivoir* OR ivory-coast* OR ivorian* OR croatia* OR cuba* OR djibouti* OR dominica* OR ecuador* OR egypt* OR united-arab-republic* OR salvador* OR equatoguinean* OR eritrea* OR estonia* OR eswatin* OR swazi* OR swati* OR ethiopia* OR fiji* OR gabon* OR gambia* OR georgia* OR ghana* OR gold-coast* OR gibraltar* OR grenad* OR guam* OR guatemala* OR guinea* OR guyan* OR guiana* OR haiti* OR hispaniola OR hondura* OR hungar* OR india* OR indonesia* OR timor* OR iran* OR iraq* OR jamaica* OR jordan* OR kazakh* OR kenya* OR north-korea* OR kosov* OR kyrgyz* OR kirghiz* OR kirgiz* OR kirghiz* OR lao OR laos OR loatian* OR latvia* OR lebanon OR lebanese OR lesoth* OR basutoland OR mosotho* OR basotho* OR liberia* OR libya* OR lithuania* OR macedonia* OR madagasca* OR malagasy* OR malawi* OR nyasaland OR malaysia* OR maldiv* OR mali OR malian OR malians OR micronesia* OR kiribati* OR marshall-island* OR marshallese OR nauru* OR mariana* OR palau* OR tuvalu* OR mauritania* OR mauritian* OR mauritius OR mexico OR mexican* OR moldov* OR mongol* OR montenegr* OR morocc* OR ifni OR mozambi* OR myanma* OR burma* OR burmese* OR namibia* OR nauruan* OR nepal* OR nicaragua* OR niger OR nigeria* OR nigerien* OR oman* OR muscat OR pakistan* OR panama* OR paraguay* OR peru OR peruvian* OR philippin* OR philipin* OR phillipin* OR phillippin* OR filipin* OR poland OR polish OR pole OR poles OR romania* OR russia* OR ussr OR soviet-union* OR union-of-soviet-socialist-republic* OR rwand* OR ruand* OR samoa* OR pacific-island* OR polynesia* OR sao-tome* OR senegal* OR serbia* OR seychell* OR sierra-leone* OR slovak* OR melanesia* OR solomon-island* OR norfolk-island* OR somali* OR sri-lanka* OR ceylon* OR saint-kitt* OR st-kitt* OR kittitian* OR saint-lucia* OR st-lucia* OR saint-vincent* OR st-vincent* OR vincentian* OR grenadin* OR sudan* OR surinam* OR syria* OR tadjik* OR tadzhik* OR tajik* OR tanzania* OR tanganyika* OR thailand* OR thai OR siam OR timor* OR togo OR togolese* OR tonga* OR trinidad* OR tobago* OR tunisia* OR turkey OR turk OR turks OR turkmen* OR uganda* OR ukrain* OR uruguay* OR uzbek* OR vanuatu* OR new-hebride* OR venezuela* OR vietnam* OR viet-nam OR viet-names* OR middle-east* OR west-bank OR gaza OR palestin* OR yemen* OR yugoslav* OR zambia* OR zimbabwe* OR rhodesia* OR global-south OR magreb* OR maghrib* OR sahara* OR west-indies OR caribbean* OR central-america* OR latin-america* OR south-america* OR central-asia* OR north-asia* OR northern-asia* OR southeastern-asia* OR south-eastern-asia* OR southeast-asia* OR south-east-asia* OR west-asia* OR western-asia* OR east-europe* OR eastern-europe*))) AND (PY=(2005-2021)) NOT (TS=((animal* OR nonhuman* OR rat OR rats OR mouse OR mice OR rodent* OR murine* OR primate* OR monkey* OR dog OR dogs OR canine* OR pig* OR porcine*) NOT (human* AND (animal* OR nonhuman* OR rat OR rats OR mouse OR mice OR rodent* OR murine* OR primate* OR monkey* OR dog OR dogs OR canine* OR pig* OR porcine*)))) NOT (TI=(editorial* OR comment* OR case-report* OR systematic-review* OR meta-analys* OR ((trial*) AND (protocol*)))) AND (LD=2021-06-01/2023-01-01)

**---**

**Embase (Scopus)
1,000 Results**

(TITLE-ABS-KEY(large-intestine* OR large-bowel* OR cecum* OR c?ecum* OR colorect* OR colon* OR rectum* OR rectal OR sigmoid* OR abdominoperineal* OR ((flexur*) AND (colon* OR spleen* OR splenic OR hepatic*)) OR ((abdomen OR abdomin*) AND (perineal*)) W/3 (neoplas* OR cancer* OR adenoma* OR carcinoma* OR adenocancer* OR adenocarcinoma* OR malignan* OR tumor* OR tumour* OR metasta* OR lesion* OR mass*)) AND TITLE-ABS-KEY(colectom* OR hemicolectom* OR proctectom* OR proctocolectom* OR coloproctectom* OR surger* OR surgical* OR resection* OR debulk* OR excision*)) AND TITLE-ABS-KEY(surviv* OR prognos* OR mortalit* OR death* OR dead OR died OR fatal* OR outcome* OR {OS} OR {DFS} OR {PFS} OR {MOS}) AND ((TITLE-ABS-KEY(afghan* OR africa* OR albania* OR algeria* OR angola* OR argentin* OR armenia* OR azerbaijan* OR bangladesh* OR bangalee* OR bajan* OR belarus* OR byelarus* OR belorus* OR byelorus* OR belize* OR benin* OR dahomey OR bhutan* OR bolivia* OR bosnia* OR herzegovin* OR botswana* OR batswana* OR bechuanaland* OR brazil* OR brasil* OR bulgaria* OR burkina* OR upper-volta OR burundi* OR urundi* OR cabo-verde* OR cape-verde* OR cambodia* OR kampuchea* OR khmer* OR cameroon* OR cameron* OR cameroun* OR ubangi* OR chad OR chadian* OR chile OR chilean* OR china OR chinese OR colombia* OR comoro* OR comorian* OR mayotte* OR congo OR congolese OR zaire* OR costa-rica* OR cote-d-ivoir* OR ivory-coast* OR ivorian* OR croatia* OR cuba* OR djibouti* OR dominica* OR ecuador* OR egypt* OR united-arab-republic* OR salvador* OR equatoguinean* OR eritrea* OR estonia* OR eswatin* OR swazi* OR swati* OR ethiopia* OR fiji* OR gabon* OR gambia* OR georgia* OR ghana* OR gold-coast* OR gibraltar* OR grenad* OR guam* OR guatemala* OR guinea* OR guyan* OR guiana* OR haiti* OR hispaniola OR hondura* OR hungar* OR india* OR indonesia* OR timor* OR iran* OR iraq* OR jamaica* OR jordan* OR kazakh* OR kenya* OR north-korea* OR kosov* OR kyrgyz* OR kirghiz* OR kirgiz* OR kirghiz* OR lao OR laos OR loatian* OR latvia* OR lebanon* lebanese OR lesoth* OR basutoland OR mosotho* OR basotho* OR liberia* OR libya* OR lithuania* OR macedonia* OR madagasca* OR malagasy* OR malawi* OR nyasaland OR malaysia* OR maldiv* OR mali OR malian OR malians OR micronesia* OR kiribati* OR marshall-island* OR marshallese OR nauru* OR mariana* OR palau* OR tuvalu* OR mauritania* OR mauritian* OR mauritius OR mexico OR mexican* OR moldov* OR mongol* OR montenegr* OR morocc* OR ifni OR mozambi* OR myanma* OR burma* OR burmese* OR namibia* OR nauruan* OR nepal* OR nicaragua* OR niger OR nigeria* OR nigerien* OR oman* OR muscat OR pakistan* OR panama* OR paraguay* OR peru OR peruvian* OR philippin* OR philipin* OR phillipin* OR phillippin* OR filipin* OR poland OR polish OR pole OR poles OR romania* OR russia* OR ussr OR soviet-union* OR union-of-soviet-socialist-republic* OR rwand* OR ruand* OR samoa* OR pacific-island* OR polynesia* OR sao-tome* OR senegal* OR serbia* OR seychell* OR sierra-leone* OR slovak* OR melanesia* OR solomon-island* OR norfolk-island* OR somali* OR sri-lanka* OR ceylon* OR saint-kitt* OR st-kitt* OR kittitian* OR saint-lucia* OR st-lucia* OR saint-vincent* OR st-vincent* OR vincentian* OR grenadin* OR sudan* OR surinam* OR syria* OR tadjik* OR tadzhik* OR tajik* OR tanzania* OR tanganyika* OR thailand* OR thai OR thais OR siam OR timor* OR togo OR togolese* OR tonga* OR trinidad* OR tobago* OR tunisia* OR turkey OR turk OR turks OR turkmen* OR uganda* OR ukrain* OR uruguay* OR uzbek* OR vanuatu* OR new-hebride* OR venezuela* OR vietnam* OR viet-nam OR viet-names* OR middle-east* OR west-bank OR gaza OR palestin* OR yemen* OR yugoslav* OR zambia* OR zimbabwe* OR rhodesia* OR global-south OR magreb* OR maghrib* OR sahara* OR west-indies OR caribbean* OR central-america* OR latin-america* OR south-america* OR central-asia* OR north-asia* OR northern-asia* OR southeastern-asia* OR south-eastern-asia* OR southeast-asia* OR south-east-asia* OR west-asia* OR western-asia* OR east-europe* OR eastern-europe* OR lmic OR lmics OR third-world OR lami-countr* OR transitional-countr* OR ((developing OR less-developed OR lesser-developed OR under-developed OR underdeveloped OR middle-income OR low-income OR lower-income OR underserved OR under-served OR deprived OR poor OR poorer) W/3 (countr* OR nation* OR population* OR world* OR econom*)) OR ((low OR lower) W/3 (gdp OR gnp OR gross-domestic OR gross-national)) OR ((emerging) W/3 (econom* OR nation*)))) OR (AFFIL(afghan* OR africa* OR albania* OR algeria* OR angola* OR argentin* OR armenia* OR azerbaijan* OR bangladesh* OR bangalee* OR bajan* OR belarus* OR byelarus* OR belorus* OR byelorus* OR belize* OR benin* OR dahomey OR bhutan* OR bolivia* OR bosnia* OR herzegovin* OR botswana* OR batswana* OR bechuanaland* OR brazil* OR brasil* OR bulgaria* OR burkina* OR upper-volta OR burundi* OR urundi* OR cabo-verde* OR cape-verde* OR cambodia* OR kampuchea* OR khmer* OR cameroon* OR cameron* OR cameroun* OR ubangi* OR chad OR chadian* OR chile OR chilean* OR china OR chinese OR colombia* OR comoro* OR comorian* OR mayotte* OR congo OR congolese OR zaire* OR costa-rica* OR cote-d-ivoir* OR ivory-coast* OR ivorian* OR croatia* OR cuba* OR djibouti* OR dominica* OR ecuador* OR egypt* OR united-arab-republic* OR salvador* OR equatoguinean* OR eritrea* OR estonia* OR eswatin* OR swazi* OR swati* OR ethiopia* OR fiji* OR gabon* OR gambia* OR georgia* OR ghana* OR gold-coast* OR gibraltar* OR grenad* OR guam* OR guatemala* OR guinea* OR guyan* OR guiana* OR haiti* OR hispaniola OR hondura* OR hungar* OR india* OR indonesia* OR timor* OR iran* OR iraq* OR jamaica* OR jordan* OR kazakh* OR kenya* OR north-korea* OR kosov* OR kyrgyz* OR kirghiz* OR kirgiz* OR kirghiz* OR lao OR laos OR loatian* OR latvia* OR lebanon OR lebanese OR lesoth* OR basutoland OR mosotho* OR basotho* OR liberia* OR libya* OR lithuania* OR macedonia* OR madagasca* OR malagasy* OR malawi* OR nyasaland OR malaysia* OR maldiv* OR mali OR malian OR malians OR micronesia* OR kiribati* OR marshall-island* OR marshallese OR nauru* OR mariana* OR palau* OR tuvalu* OR mauritania* OR mauritian* OR mauritius OR mexico OR mexican* OR moldov* OR mongol* OR montenegr* OR morocc* OR ifni OR mozambi* OR myanma* OR burma* OR burmese* OR namibia* OR nauruan* OR nepal* OR nicaragua* OR niger OR nigeria* OR nigerien* OR oman* OR muscat OR pakistan* OR panama* OR paraguay* OR peru OR peruvian* OR philippin* OR philipin* OR phillipin* OR phillippin* OR filipin* OR poland OR polish OR pole OR poles OR romania* OR russia* OR ussr OR soviet-union* OR union-of-soviet-socialist-republic* OR rwand* OR ruand* OR samoa* OR pacific-island* OR polynesia* OR sao-tome* OR senegal* OR serbia* OR seychell* OR sierra-leone* OR slovak* OR melanesia* OR solomon-island* OR norfolk-island* OR somali* OR sri-lanka* OR ceylon* OR saint-kitt* OR st-kitt* OR kittitian* OR saint-lucia* OR st-lucia* OR saint-vincent* OR st-vincent* OR vincentian* OR grenadin* OR sudan* OR surinam* OR syria* OR tadjik* OR tadzhik* OR tajik* OR tanzania* OR tanganyika* OR thailand* OR thai OR siam OR timor* OR togo OR togolese* OR tonga* OR trinidad* OR tobago* OR tunisia* OR turkey OR turk OR turks OR turkmen* OR uganda* OR ukrain* OR uruguay* OR uzbek* OR vanuatu* OR new-hebride* OR venezuela* OR vietnam* OR viet-nam OR viet-names* OR middle-east* OR west-bank OR gaza OR palestin* OR yemen* OR yugoslav* OR zambia* OR zimbabwe* OR rhodesia* OR global-south OR maghreb* OR maghrib* OR sahara* OR west-indies OR caribbean* OR central-america* OR latin-america* OR south-america* OR central-asia* OR north-asia* OR northern-asia* OR southeastern-asia* OR south-eastern-asia* OR southeast-asia* OR south-east-asia* OR west-asia* OR western-asia* OR east-europe* OR eastern-europe*))) AND (PUBYEAR AFT 2004) AND (INDEX(embase)) AND NOT (KEY((animal* OR nonhuman* OR rat OR rats OR mouse OR mice OR rodent* OR murine* OR primate* OR monkey* OR dog OR dogs OR canine* OR pig* OR porcine*) AND NOT (human* AND (animal* OR nonhuman* OR rat OR rats OR mouse OR mice OR rodent* OR murine* OR primate* OR monkey* OR dog OR dogs OR canine* OR pig* OR porcine*)))) AND NOT (TITLE(editorial* OR comment* OR case-report* OR systematic-review* OR meta-analys* OR ((trial*) AND (protocol*)))) AND (ORIG-LOAD-DATE AFT 1622566800)

**---**

**GIM
248 Results***with publication date limit of 2021-2023*

(tw:(( large-intestine* OR large-bowel* OR cecum* OR coecum* OR caecum* OR colorect* OR colon* OR rectum* OR rectal OR sigmoid* OR abdominoperineal OR splenic-flexur* OR colon-flexur* OR colonic-flexur* OR hepatic-flexur* OR abdominal-perineal* OR abdominoperineal* OR abdomen-perineal*) AND (neoplas* OR cancer* OR adenoma* OR carcinoma* OR adenocarcinoma* OR malignan* OR tumor* OR tumour* OR metasta* OR lesion* OR mass*))) AND (tw:(colectom* OR hemicolectom* OR proctectom* OR proctocolectom* OR coloproctectom* OR surger* OR surgical* OR resection* OR debulk* OR excision*)) AND (tw:(surviv* OR prognos* OR mortalit* OR death* OR dead OR died OR fatal* OR outcome* OR OS OR DFS OR PFS OR MOS)) AND NOT (mh:(Animals AND NOT (Humans AND Animals))) AND NOT (mh:(Editorial OR Comment OR clinical-trial-protocol)) AND NOT (ti:(case-report*OR systematic-review* OR meta-analys*))
